# Zengwei Chengqi Decoction Reduces Inflammation in Acute Intestinal Obstruction

**DOI:** 10.1155/mi/8826129

**Published:** 2026-03-13

**Authors:** Ying Gong, Zi-Hua Xuan, Yue Dong, Chun-Bin Zhang, Yu-Chen Liu, Jin-Hui Zhang, Qing Li, Cong-Bin Liu, Dong-Mei Xie

**Affiliations:** ^1^ College of Pharmacy, Anhui University of Chinese Medicine, No. 350 Longzihu Road, Xinzhan District, Hefei, 230012, Anhui, China, ahtcm.edu.cn; ^2^ Chuzhou Integrated Traditional Chinese and Western Medicine Hospital, Chuzhou, 239000, China

**Keywords:** animal experiment, inflammatory factors, intestinal obstruction, multi-target mechanism, network pharmacology, Zengwei Chengqi Decoction

## Abstract

**Objective:**

This study investigates the therapeutic effects of Zengwei Chengqi Decoction (ZW) on acute intestinal obstruction (AIO), focusing on its ability to alleviate inflammation and reduce associated tissue damage. Network pharmacology and experimental animal models were used to explore the mechanisms of action.

**Methods:**

Using network pharmacology, we identified 228 active compounds in ZW and 420 corresponding targets linked to inflammation. Gene Ontology (GO) and Kyoto Encyclopedia of Genes and Genomes (KEGG) enrichment analyses were used to identify the biological pathways influenced by ZW. In vivo, an SD rat model of AIO was induced, and the therapeutic effects of ZW were assessed by histological examination of tissue damage and serum cytokine levels.

**Results:**

Treatment with ZW significantly mitigated tissue damage in AIO rats. Additionally, serum levels of pro‐inflammatory cytokines, including IL‐1β, IL‐6, and TNF‐α, were substantially reduced in the ZW‐treated group compared to the control group (*p* < 0.01).

**Conclusion:**

ZW shows strong potential for modulating inflammation in AIO, as evidenced by reduced cytokine levels and improved tissue integrity. These findings provide valuable insights into the mechanisms underlying its therapeutic effects, positioning ZW as a promising treatment for AIO.

## 1. Introduction

Acute intestinal obstruction (AIO) is a common and potentially life‐threatening gastrointestinal emergency characterized by partial or complete obstruction of the flow of nutrients and fluids within the intestines [1–3]. Symptoms include pain, bloating, nausea, vomiting, and an inability to eat [4–6]. The clinical presentation can vary significantly, ranging from mild discomfort to severe physiological stress [7]. In traditional medicine, a modified formula based on the “Dachengqi Decoction” from Zhang Zhongjing’s *Shanghan Lun* is often used for treatment, showing significant therapeutic effects [8, 9]. To enhance the efficacy and make the treatment milder for elderly patients, a major hospital in Anhui Province developed a clinical prescription based on Dachengqi Decoction for treating adhesive intestinal obstruction, adjusting the dosage through repeated diagnostic differentiation. Combined with nonsurgical treatment, the self‐made herbal formula (hereinafter referred to as “the prescription”) is used as the standard intervention, leading to favorable clinical outcomes with significant improvements in the treatment period and patients’ pain levels. However, due to the complexity of the formula’s components and the unclear mechanisms of action, it is of great scientific significance to conduct further research on its independent efficacy and underlying mechanisms.

The use of traditional Chinese Medicine (TCM) formulas to treat intestinal obstruction is characterized by multiple components, multiple targets, and multiple pathways [10–12]. Network pharmacology is an effective analytical method that integrates the interactions between multiple drug components and disease pathways, offering a global perspective [13]. This approach has been widely applied in TCM, particularly for multicomponent, multitarget therapeutic strategies. For example, network pharmacology has been used to identify key active components and targets in TCM formulas, thereby elucidating their potential mechanisms of action [14]. Therefore, network pharmacology can be employed to analyze the components of this formula, study its mechanisms of action, and validate its pharmacological effects in treating intestinal obstruction through animal experiments. Animal models are essential for evaluating drug efficacy, especially in studying diseases like intestinal obstruction [15, 16]. Researchers have used network pharmacology to explore the efficacy of formulas, with studies indicating that these formulas improve intestinal obstruction symptoms by regulating immune‐inflammatory responses, apoptosis, and other mechanisms [17]. The rat model of intestinal obstruction established through surgical intervention can simulate inflammatory damage, allowing for the validation of the network pharmacology screening results. Combining network pharmacology with animal experiments provides a more comprehensive understanding of the mechanisms and efficacy of the drug.

Although network pharmacology has made significant progress in studying TCM, research on the mechanisms of specific TCM formulas for treating AIO is still in its early stages. TCM formulas often contain multiple components, and their mechanisms of action are complex, making it difficult for conventional methods to fully reveal their pathways. Network pharmacology, by integrating information on drug components, targets, and pathways, provides a new perspective and methodology for studying multicomponent, multitarget drugs. In particular, although studies have revealed the anti‐inflammatory effects of some components of Zengwei Chengqi Decoction (ZW) [7], its synergistic effects involving multiple components and targets still require further systematic investigation.

This study aims to clarify the mechanisms of ZWCD in treating AIO through network pharmacology and animal experiments. Network pharmacology was used to identify active compounds, targets, and pathways via Gene Ontology (GO) and Kyoto Encyclopedia of Genes and Genomes (KEGG) analyses. In vivo validation was conducted using two complementary models: a surgically induced AIO model in Sprague‐Dawley (SD) rats and a lipopolysaccharide (LPS)‐induced intestinal obstruction/inflammation supplementary model in BALB/c mice. Serum and tissue levels of inflammatory cytokines, including interleukin‐1β (IL‐1β), interleukin‐6 (IL‐6), and tumor necrosis factor‐α (TNF‐α). The findings provide systematic evidence for the multitarget actions of ZWCD and offer novel insights and potential therapeutic targets for improving the treatment and prognosis of AIO.

## 2. Materials and Methods

### 2.1. Experimental Animals

This study strictly adheres to the relevant policies on experimental and clinical research outlined in the *Basic and Clinical Pharmacology and Toxicology* guidelines and complies with the *Regulations for the Management of Experimental Animals* and the *Animal Experiment Ethical* Guidelines. All experimental protocols were approved by the Animal Ethics Committee of our hospital (Ethics Approval No: 2024010). A total of 65 SPF‐grade healthy experimental animals were used in this study, including 35 SD rats (200–250 g, half male and half female) for establishing the surgical AIO model and 30 male BALB/c mice (22–25 g) for establishing the LPS‐induced supplementary model of inflammatory response to mimic the inflammation‐related mechanisms of intestinal obstruction. All experimental animals were provided by Jinan Pengyue Experimental Animal Breeding Co., Ltd. (License No: SCXK (Lu) 20190003).

The animals were housed individually in standard laboratory animal rooms, with the environment temperature controlled at (22 ± 2)°C, relative humidity maintained at (50 ± 5)%, and a 12‐h light/dark cycle. The animals had free access to food and water, with daily feeding to ensure adequate food and water supplies. The cages were cleaned regularly, and bedding was changed every 2 days to maintain a clean environment. Before the start of the experiment, all rats were acclimated for 7 days to reduce the impact of environmental changes on the experimental results.

### 2.2. Drugs and Instruments

The drugs used in this study include clinical experience‐based decoctions provided by Chuzhou Integrated Traditional Chinese and Western Medicine Hospital, with high, medium, and low doses corresponding to crude drug concentrations of 1.23, 0.615, and 0.3075 g/mL, respectively. Additionally, the following reagents and solutions were used during the experiment: octreotide injection (H220907, Chengdu Shengnuo Biopharmaceutical Co., Ltd.), cefemizole sodium powder for injection (H20211229, Shandong Luoxin Pharmaceutical Co., Ltd.), 5% glucose sodium chloride injection (H22050610, Anhui Shuanghe Pharmaceutical Co., Ltd.), vitamin C injection (H22062932, Chenxin Pharmaceutical Co., Ltd.), vitamin B6 injection (H22120310, China Resources Shuanghe Limin Pharmaceutical (Jinan) Co., Ltd.), potassium chloride injection (H21120543, China Resources Shuanghe Limin Pharmaceutical (Jinan) Co., Ltd.), uratan (20160920, Sinopharm Chemical Reagent Co., Ltd.), 0.9% sodium chloride injection (C23090703, Jiangxi Kelun Pharmaceutical Co., Ltd.), 4% paraformaldehyde (71032700, Beijing Labgic Technology Co., Ltd.), Glutaraldehyde (G105905, Shanghai Aladdin Biochemical Technology Co., Ltd.), osmium tetroxide (O302601, Shanghai Aladdin Biochemical Technology Co., Ltd.), acetone (H111149, Shanghai Aladdin Biochemical Technology Co., Ltd.), and epoxy resin (A758810, Shanghai Aladdin Biochemical Technology Co., Ltd.). The enzyme‐linked immunosorbent assay (ELISA) method was used to measure the levels of inflammatory factors, including IL‐1β, IL‐6, and TNF‐α, using specific ELISA kits (JL2023120001, JL2023110001, JL2023090001, Hefei NuoZhuo Biotechnology Co., Ltd.). The main instruments used in this study include the YP‐5102 electronic analytical balance (Shanghai Guangzheng Medical Equipment Co., Ltd.) and the DHG‐9140A electric constant temperature blast drying oven (Shanghai Jinghong Experimental Equipment Co., Ltd.). All experimental procedures were strictly conducted according to the equipment operation guidelines to ensure the accuracy and reproducibility of the experimental data.

### 2.3. Network Pharmacology Databases and Analytical Methods

Multiple public databases and bioinformatics platforms were used to investigate the mechanisms of ZWCD in treating intestinal obstruction. Active compounds and targets were retrieved from the TCM Systems Pharmacology Database and Analysis Platform (TCMSP) (https://old.tcmsp-e.com/tcmsp.php); compound structures from PubChem (https://pubchem.ncbi.nlm.nih.gov). Targets were predicted using SwissTargetPrediction and PharmMapper. Disease‐related genes were collected from GeneCards (https://www.genecards.org/) and DisGeNET (https://www.disgenet.org/); overlapping targets were identified with Venny 2.1.0. A protein–protein interaction (PPI) network was constructed via STRING. GO and KEGG enrichment analyses were performed using Metascape. Protein structures were obtained from the RCSB Protein Data Bank (https://www.rcsb.org/); molecular docking was conducted to evaluate binding affinities between key targets and core compounds.

### 2.4. Study Participants

This study randomly selected 100 patients with acute adhesive intestinal obstruction who met the inclusion criteria from the hospital database. The patients were divided into the Observation group (*n* = 50) and the Control group (*n* = 50) using a random number table. This study strictly followed the requirements outlined in the *Helsinki Declaration* and the *Ethical Guidelines for Biomedical Research Involving Human Participants*. All patients signed informed consent forms, and the study protocol was approved by the hospital’s Ethics Committee.

#### 2.4.1. Inclusion Criteria

(1) Diagnosis of acute adhesive intestinal obstruction according to the diagnostic criteria in the *Clinical Practice Guidelines for Surgery*; (2) Age between 18 and 75 years; (3) Stable condition, clear consciousness, able to cooperate with treatment and follow‐up; (4) No other medications affecting intestinal function within the last month; (5) Voluntary participation in the study and signing of the informed consent form.

#### 2.4.2. Exclusion Criteria

(1) Diagnosis of complete strangulated intestinal obstruction or patients requiring emergency surgery; (2) Presence of severe comorbidities (e.g., severe liver or kidney dysfunction, cardiovascular diseases, cerebrovascular diseases); (3) Pregnant or breastfeeding women; (4) Patients with cognitive impairments who are unable to cooperate with the study or express subjective feelings; (5) Previous interventions that may affect the study results; (6) Poor compliance or patients unable to complete follow‐up assessments.

### 2.5. Treatment Methods

The control group patients received conventional Western medical treatments, including gastrointestinal decompression, fluid resuscitation, anti‐infection therapy, and correction of electrolyte and acid‐base imbalances. The observation group received ZW treatment in addition to the conventional therapy. Both groups were treated for 7 days, during which clinical indicators were monitored daily, and treatment outcomes were assessed. The specific treatment protocols are shown in Figure S1.

Control group (conventional western treatment): (1) gastrointestinal Decompression: all patients were fasted and had their gastric contents continuously drained via a gastric tube under negative pressure; (2) fluid resuscitation: intravenous fluids were administered to maintain electrolyte balance, including daily infusion of 100 mL of 0.9% sodium chloride injection (intravenous drip) and a mixture of 1 g of 5% glucose sodium chloride injection + 1 g of vitamin C + 0.1 g of vitamin B6 + 1 g of potassium chloride (intravenous drip); (3) anti‐infection and gastrointestinal function regulation: cefemizole sodium (dissolved in 0.9% sodium chloride solution, 100 mL/day, intravenous drip); Octreotide (0.1 mg, subcutaneous injection every 8 h).

Observation group (ZW combined treatment): in addition to the treatments provided to the Control group, the Observation group patients received ZW. The formulation was prepared by the hospital’s chief pharmacist, with the daily dosage concentrated to 100 mL and maintained at 40°C, administered via a gastric tube. It was removed after 90 min of gastric tube placement, and the color and drainage volume of gastric contents were closely observed. 2 h after drug administration, a 200 mL decoction of Da Huang (DH) was administered via enema, retained for 60 min, and then expelled. This was performed twice daily for 7 days [18].

All patients underwent 7 days of treatment, with daily recording of clinical symptom changes, inflammatory factor levels, and imaging assessments. The results were comprehensively compared between the two groups in the results section.

### 2.6. Observation Indicators

Clinical and biochemical indicators were used to evaluate treatment effects in the two patient groups. The primary observation indicators include urinary amylase (Urinary AMY) recovery time and length of hospitalization, which are used to assess the recovery of the patient’s condition. All patients underwent biochemical tests before and after treatment, with the white blood cell count (WBC) and C‐reactive protein (CRP) levels recorded to reflect the overall inflammatory status. Additionally, the treatment effects of the two groups were comprehensively assessed based on the patient’s clinical symptoms.

### 2.7. Efficacy Evaluation Criteria

Efficacy evaluation was based on the standards outlined in *Practical Diagnosis and Treatment of Integrative Traditional Chinese and Western Medicine*, with treatment outcomes classified into four categories: (1) Cure: Clinical symptoms completely disappear, there is no discomfort upon abdominal palpation, obstruction is resolved, bowel sounds return to normal, and the passage of intestinal gas and stool is smooth. (2) Significant Effect: Clinical symptoms are largely resolved, with no nausea or vomiting, normal bowel sounds, and smooth passage of intestinal gas and stool. (3) Effective: Clinical symptoms are partially relieved, though not completely resolved, and there is no significant impact on normal eating and bowel movements. (4) Ineffective: After 7 days of treatment, there is no significant improvement in clinical symptoms, or the condition worsens. Efficacy assessment for all patients was performed independently by two clinical physicians to ensure objectivity and consistency.

### 2.8. Construction and Screening of the Component Library

The active component library of ZW was established based on the TCMSP database and relevant literature. The formula consists of Zhi Shi (ZS), Hou Po (HP), Mu Xiang (MX), Chi Shao (CS), Tao Ren (TR), Dan Shen (DS), Jin Yin Hua (JYH), Huang Qin (HQ), Huo Ma Ren (HMR), DH, and Mang Xiao (MGX). The screening criteria for components included oral bioavailability (OB) ≥30% and drug‐likeness (DL) ≥0.18, with further verification through the PubChem and ChemSpider databases. The selected active components were cross‐referenced with literature and validated using the methods from Zhang et al. [19] to ensure their biological activity and pharmacological validity. Ultimately, a component database for ZW was established, providing data support for subsequent network pharmacology analysis and molecular docking studies.

### 2.9. Screening and Analysis of Intersecting Targets in Inflammatory Diseases

Potential targets of the active components in ZWCD were predicted using the SwissTargetPrediction and PharmMapper databases. Inflammatory disease‐related gene targets were retrieved from GeneCards and DisGeNET. To improve accuracy, duplicates and irrelevant entries were removed. The remaining compound targets were compared with disease‐related targets using the Venny 2.1.0 online tool to identify intersecting targets, which were subsequently used for network pharmacology analysis to explore the regulatory mechanisms of ZWCD in inflammatory diseases.

### 2.10. Construction of TCM Component‐Target Network

A component‐target network of ZWCD was constructed based on the identified active components and their predicted targets. Pairing analysis was used to extract common active ingredients and generate a component list. The herbal compounds, individual components, common components, and their corresponding target genes were imported into Cytoscape 3.9.1. Topological parameters, including Degree, Betweenness Centrality, and Closeness Centrality were calculated using the CytoNCA plugin to evaluate node importance. Key active components and core targets were identified according to Degree values, and a component‐target network diagram was generated to visualize the multicomponent and multitarget characteristics of ZWCD.

### 2.11. Construction of PPI Network

The STRING database (https://string-db.org/) was used to construct the PPI network of the targets of ZWCD. Intersecting targets were entered into STRING with the species set to *Homo sapiens* and the minimum required interaction score set to ≥0.4 to obtain reliable PPI data. The interaction data were exported and imported into Cytoscape 3.9.1 for visualization. The CytoNCA plugin was applied to calculate topological parameters, including Degree, Betweenness Centrality, and Closeness Centrality, to evaluate node importance. Core targets were then identified based on Degree values, and the PPI network was established to support further elucidation of the mechanisms of ZWCD.

### 2.12. GO and KEGG Pathway Enrichment Analysis

The Metascape database (https://metascape.org/) was used to perform GO and KEGG pathway enrichment analyses of the intersecting targets to explore the potential mechanisms of ZWCD. GO analysis covered biological processes (BP), cellular components (CC), and molecular functions (MF), while KEGG analysis identified key signaling pathways related to inflammatory diseases. Enrichment was conducted with a significance threshold of *p* < 0.05, and the top 20 pathways were extracted based on ascending *p*‐values. A bubble plot was generated using the microbiome platform to visualize GO and KEGG results. Component‐target‐pathway association data were then imported into Cytoscape 3.9.1, where a signaling pathway regulatory network was constructed to illustrate the multi‐pathway synergistic actions of ZWCD.

### 2.13. Molecular Docking

Molecular docking was performed to evaluate the interactions between active components of ZWCD and core targets. Three‐dimensional PDB structures of key targets were downloaded from the RCSB PDB database (https://www.rcsb.org/), with water molecules and ligands removed using PyMOL 2.5 followed by structural optimization and energy minimization. Molecular structures of the main active components were obtained in mol2 format from the TCMSP database, then converted and optimized using OpenBabel 3.1.1. Polar hydrogen atoms were added, Gasteiger charges calculated, and grid boxes defined to cover target active sites with a grid spacing of 0.375 Å using AutoDockTools 1.5.7. Docking was conducted with AutoDock Vina 1.2.3, and conformations with binding energy ≤−1.2 kcal/mol were selected. Docking results were visualized in PyMOL 2.5, highlighting hydrogen bonds, hydrophobic interactions, and other ligand‐receptor interactions to provide structural insights into the mechanisms of ZWCD.

### 2.14. Establishment of the Surgical AIO Rat Model

An AIO model was established in rats with reference to the literature [20, 21]. Rats were fasted for 12 h with free access to water and anesthetized by intraperitoneal injection of 20% urethane (5 mL/kg). In the sham group, the mesentery was pierced without intestinal ligation; the control group received no surgical intervention. Postoperatively, bedding was changed and aseptic conditions maintained to prevent infection.

At 24 h, five rats from both the model and sham groups were randomly selected to assess modeling success. Fecal analysis showed markedly reduced fecal output in the model group (≈30% of controls). An intestinal transit test was performed by gavage with carbon suspension; after 20 min, the intestine from pylorus to cecum was excised, total length (L1) and carbon transit distance (L2) were measured, and transit rate was calculated (*D* = L2/L1 × 100%). A significant reduction in transit rate indicated impaired motility. Histopathology of the ileocecal region revealed inflammatory cell infiltration, vascular congestion, stromal edema, epithelial disorganization, and villus loss, further confirming successful modeling.

### 2.15. Grouping and Drug Administration

Following the principles of randomization and balance, SD rats were randomly assigned using a computer‐generated random number table into seven groups (*n* = 5 per group): control, model, sham‐operated, positive drug, low‐dose, medium‐dose, and high‐dose groups. Rats in the positive drug group received octreotide (50 μg/kg) by intraperitoneal injection. Rats in the experimental groups were administered ZWCD solution twice daily for 10 consecutive days by gavage combined with enema. The dosage was calculated as 1 mL/100 g body weight, with high, medium, and low doses determined from the human equivalent dose using the body surface area conversion method, corresponding to 20, 10, and 5 mL/(kg d), respectively [22–24]. Rats in the control, model, and sham‐operated groups were gavaged with equal volumes of normal saline. During the experimental process, all rats were raised and managed under standardized conditions to ensure that each group received treatment under identical circumstances. Standardized perioperative management was applied to all rats, including strict aseptic procedures (autoclaving of instruments, triple disinfection of the surgical field with povidone‐iodine), postoperative wound care and application of antibiotic ointment, daily replacement of sterile bedding, as well as provision of high‐protein feed and electrolyte‐containing drinking water. For rats with feeding difficulties, nutritional support via gavage was provided to reduce the risk of postoperative infection and malnutrition.

### 2.16. LPS‐Induced Mouse Inflammation Supplementary Model

A total of 30 SPF‐grade male BALB/c mice (22–25 g) were randomly divided into six groups (*n* = 5 per group): Control group (sham surgery group, laparotomy without establishing the intestinal obstruction model), LPS group (LPS‐induced inflammation/intestinal obstruction model group without drug intervention), LPS + Octreotide group (positive control, treated with octreotide 50 μg/kg), LPS + Luteolin group (LPS‐induced followed by Luteolin 10 μg/kg) [25], LPS + Stigmasterol group (LPS‐induced followed by Stigmasterol 50 mg/kg) [26], and LPS + Beta‐sitosterol group (LPS‐induced followed by Beta‐sitosterol 50 mg/kg) [27]. Grouping was performed using a computer‐generated random number table to ensure scientific rigor and reproducibility. Prior to modeling, the mice were fasted for 12 h with free access to water and anesthetized with an intraperitoneal injection of 2% sodium pentobarbital (40 mg/kg). The model and drug treatment groups were induced by intraperitoneal injection of LPS (2%, 5 mL/kg), while the Control group underwent laparotomy only without LPS injection. After the procedure, the mice were allowed free access to food and water. Experimental evaluations were conducted 48 h post‐induction to assess intestinal tissue damage, inflammatory factor levels, and signaling pathway activation.

### 2.17. Western Blot Experiment

Western blot analysis was performed to detect the expression of MAPK1/MAPK3 and their phosphorylated forms (p‐MAPK1/p‐MAPK3), as well as total AKT, phosphorylated AKT, and PI3K, in intestinal tissues of LPS‐induced BALB/c mice. After 24 h of treatment, total protein was extracted from intestinal tissues, quantified using the BCA method, separated by SDS‐PAGE, and transferred onto PVDF membranes. Membranes were blocked for 1 h and incubated overnight at 4°C with primary antibodies against MAPK1/MAPK3 (1:1000, Sangon Biotech, D151753), p‐MAPK1/MAPK3 (1:1000, Abclonal, A16736), AKT (1:5000, GeneTex, GTX121937), p‐AKT (1:2000, Proteintech, 66444‐1‐Ig), PI3K (1:200, Proteintech, 20584‐1‐AP), and GAPDH (1:2000, Proteintech, 60004‐1‐Ig). The following day, membranes were incubated with HRP‐conjugated secondary antibodies for 1 h, and protein bands were visualized using an ECL detection system. Band intensity was quantified using ImageJ software, and MAPK1/MAPK3, AKT, and PI3K expression levels were normalized to GAPDH.

### 2.18. Quantitative Polymerase Chain Reaction (qPCR) Experiment

Real‐time qPCR was performed to measure the mRNA expression levels of MAPK1 and MAPK3 in intestinal tissues of LPS‐induced BALB/c mice in order to assess whether the drug affects gene transcription. The experimental groups were the same as those in the Western blot experiment. After 24 h of cell treatment, total RNA was extracted, and RNA purity was assessed using a NanoDrop spectrophotometer (260/280 ratio between 1.8 and 2.0). The RNA was then reverse transcribed into cDNA using a reverse transcription kit. The qPCR reaction system consisted of 20 μL, including SYBR Green reagent, primers, and cDNA template. The amplification conditions were as follows: 95°C for 2 min for predenaturation, followed by 40 cycles of 95°C for 15 s, and 60°C for 30 s. The primer sequences were MAPK1‐F: 5′‐TGCTGAAGCGCCATTCAAGT‐3′, MAPK1‐R: 5′‐AGACGAAGCCTAACATCCTCA‐3′, MAPK3‐F: 5′‐TGCGATTCCGCCATGAGAAT‐3′, and MAPK3‐R: 5′‐GGTCGCAGGTGGTGTTGATA‐3′. GAPDH was used as the reference gene, and the relative gene expression was calculated using the ΔΔCt method.

### 2.19. ELISA Experiment

ELISA was performed to measure IL‐1β, IL‐6, and TNF‐α levels in rat serum and mouse serum. For animal experiments, blood was collected (rat: from the abdominal aorta 1 h after final administration; mouse: 48 h post‐surgery), centrifuged at 3000 rpm for 15 min, and serum was stored at −20°C. For cell experiments, supernatants were collected after 24 h of treatment. ELISA kits were used according to the manufacturer’s instructions. Standards and samples were added to 96‐well plates, incubated at 37°C for 1 h, washed five times, and followed by incubation with enzyme‐labeled secondary antibodies for 30 min. The reaction was terminated, and absorbance was measured at 450 nm to calculate cytokine concentrations.

### 2.20. Histological Analysis (H&E Staining)

Histological analysis of intestinal tissues (rat cecum and mouse ileocecal region) was performed using H&E staining. Tissues were collected, fixed in 4% paraformaldehyde for 24 h, dehydrated through a graded ethanol series, cleared in xylene, and embedded in paraffin. Sections (4–5 μm) were cut using a microtome, deparaffinized, rehydrated, and stained with hematoxylin for 5 min, differentiated with acid ethanol, counterstained with eosin for 2 min, dehydrated, and mounted with neutral resin. Tissue morphology, including epithelial injury, crypt architecture, inflammatory cell infiltration, and edema, was examined under a light microscope (× 100−400), and representative images were captured.

### 2.21. Transmission Electron Microscopy (TEM) Analysis

Ileocecal mucosal tissues (~1 mm^3^) were collected from each group of rats after sacrifice. Samples were fixed in 2.5% glutaraldehyde and post‐fixed in 1% osmium tetroxide, followed by graded acetone dehydration and epoxy resin embedding. Ultrathin sections (50–70 nm) were prepared, stained with uranyl acetate and lead citrate, and examined under a transmission electron microscope to evaluate ultrastructural changes in intestinal epithelial cells, tight junctions, mitochondria, and endoplasmic reticulum [28].

### 2.22. Statistical Analysis Methods

Data analysis was performed using SPSS 26.0 software for statistical processing, and GraphPad Prism 9.0.0 software was used for data visualization. The data from each group were analyzed using one‐way analysis of variance (ANOVA), and intergroup differences were considered statistically significant at *p* < 0.05. All data are presented as mean ± standard deviation (mean ± SD) to ensure the scientific accuracy and reliability of the data analysis.

## 3. Results

### 3.1. Baseline Characteristics and Efficacy Comparison of the Two Groups of Patients

The baseline characteristics of the two groups were comparable (Table S1). The Observation group included 32 males (64.0%) and 18 females (36.0%), while the Control group included 33 males (66.0%) and 17 females (34.0%). The mean age was (51.7 ± 6.4) years in the Observation group and (52.2 ± 5.9) years in the Control group, with no significant difference (*p* > 0.05). The disease duration was (1.4 ± 0.5) months and (1.1 ± 0.3) months in the two groups, respectively, also without significant difference (*p* > 0.05). Efficacy analysis (Figure 1A) showed that the length of hospital stay and urinary AMY recovery time were significantly shorter in the Observation group than in the Control group (*p* < 0.05). The overall effective rate was also higher in the Observation group (*p* < 0.05). Regarding inflammation‐related indicators (Figure 1B), WBC and hs‐CRP levels decreased significantly in both groups after treatment compared with before treatment (*p* < 0.05). There were no differences between groups before treatment (*p* > 0.05), but posttreatment levels were significantly lower in the Observation group (*p* < 0.05). In summary, the Observation group demonstrated superior outcomes in hospital stay, urinary AMY recovery, overall efficacy, and inflammatory marker reduction compared with the Control group.

Figure 1Baseline characteristics and efficacy evaluation of the two groups of patients. (A) Comparison of hospital stay and urinary AMY recovery time, showing the length of hospitalization and urinary AMY recovery time in the Observation and Control groups. Data are presented as Mean ± SD. Intergroup comparisons were performed using an independent samples *t*‐test, with  ^∗^ indicating statistical significance (*p* < 0.05). (B) Changes in inflammation‐related indicators (WBC count and hs‐CRP levels), comparing the pre‐ and posttreatment changes in WBC and hs‐CRP levels in both groups. Comparisons before and after treatment were conducted using paired *t*‐tests, and intergroup comparisons were performed using independent samples *t*‐test, with  ^∗^ indicating statistical significance (*p* < 0.05).

**Figure:**
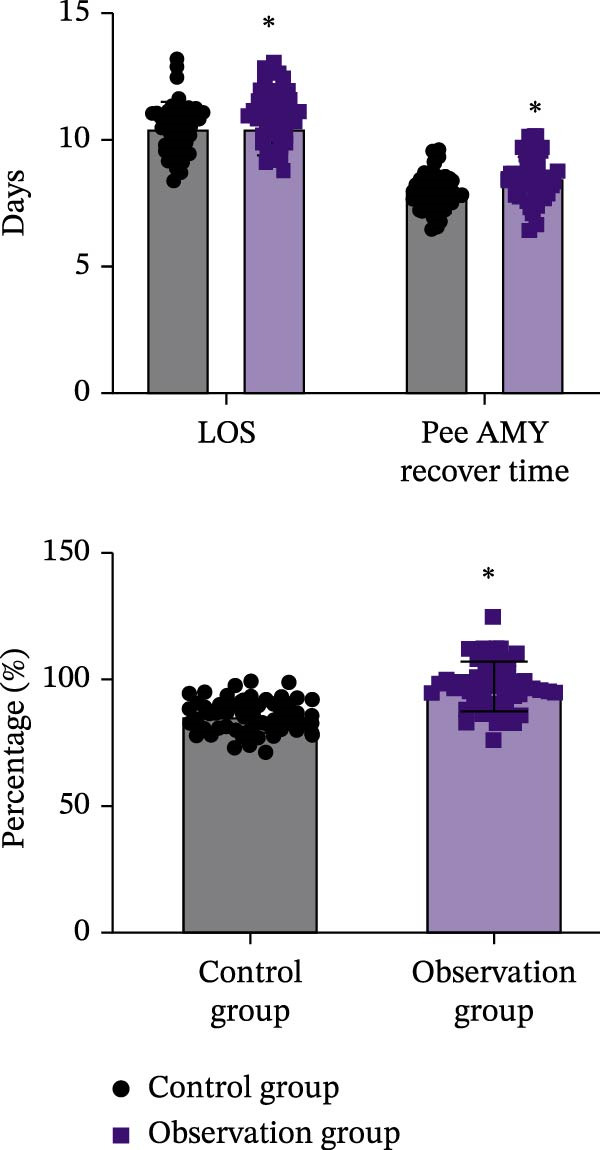
(A)

**Figure:**
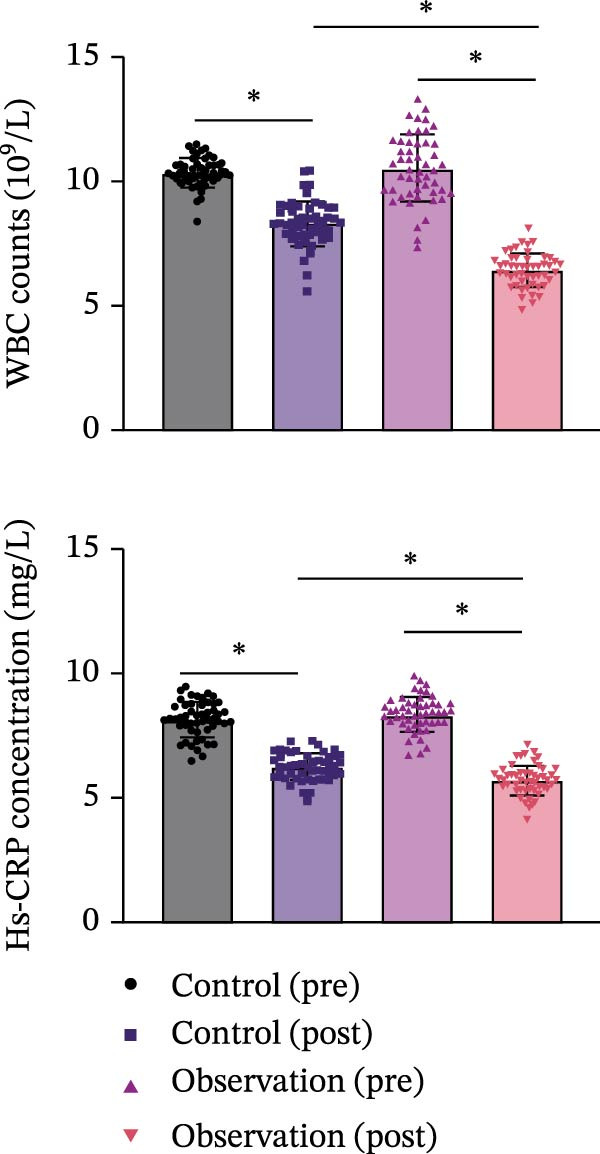
(B)

### 3.2. Network Pharmacology Analysis of ZW and Identification of Core Targets

This study systematically analyzed the chemical components, potential targets, and mechanisms of action of ZWCD. Among the 11 constituent medicines, Mangxiao (Na_2_SO_4_ · 10H_2_O) had no relevant compound information, while the other 10 yielded a total of 228 active compounds: CS 29, DH 16, DS 65, HP 2, HMR 6, HQ 36, JYH 23, MX 6, TR 23, and ZS 22. After removing duplicates, 996 unique potential gene targets were obtained. From the GeneCards and DisGeNET databases, 2461 intestinal obstruction‐related targets were retrieved, and intersection analysis identified 420 common targets (Table 1, Figure 2A).

Figure 2Compound‐target‐disease network and PPI analysis of Zengwei Chengqi Decoction. (A) Venn diagram showing the overlap between compound targets of ZWCD (blue) and disease‐related genes for intestinal obstruction (yellow); the intersecting area (gray) represents shared targets. (B) Compound‐target network. Circular nodes represent compounds from different herbs, with node colors indicating herb categories; green inverted triangles indicate shared compounds across multiple herbs; yellow squares represent targets. Node size is proportional to the Degree value. (C) PPI network. Node size and color depth are proportional to the Degree value, with colors ranging from yellow to red to indicate increasing node centrality. Red nodes highlight the most central targets.

**Figure:**
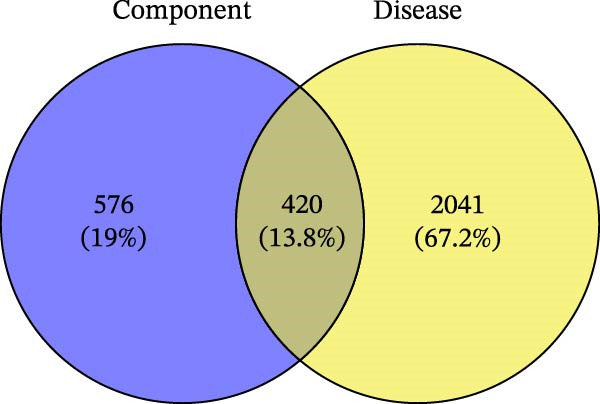
(A)

**Figure:**
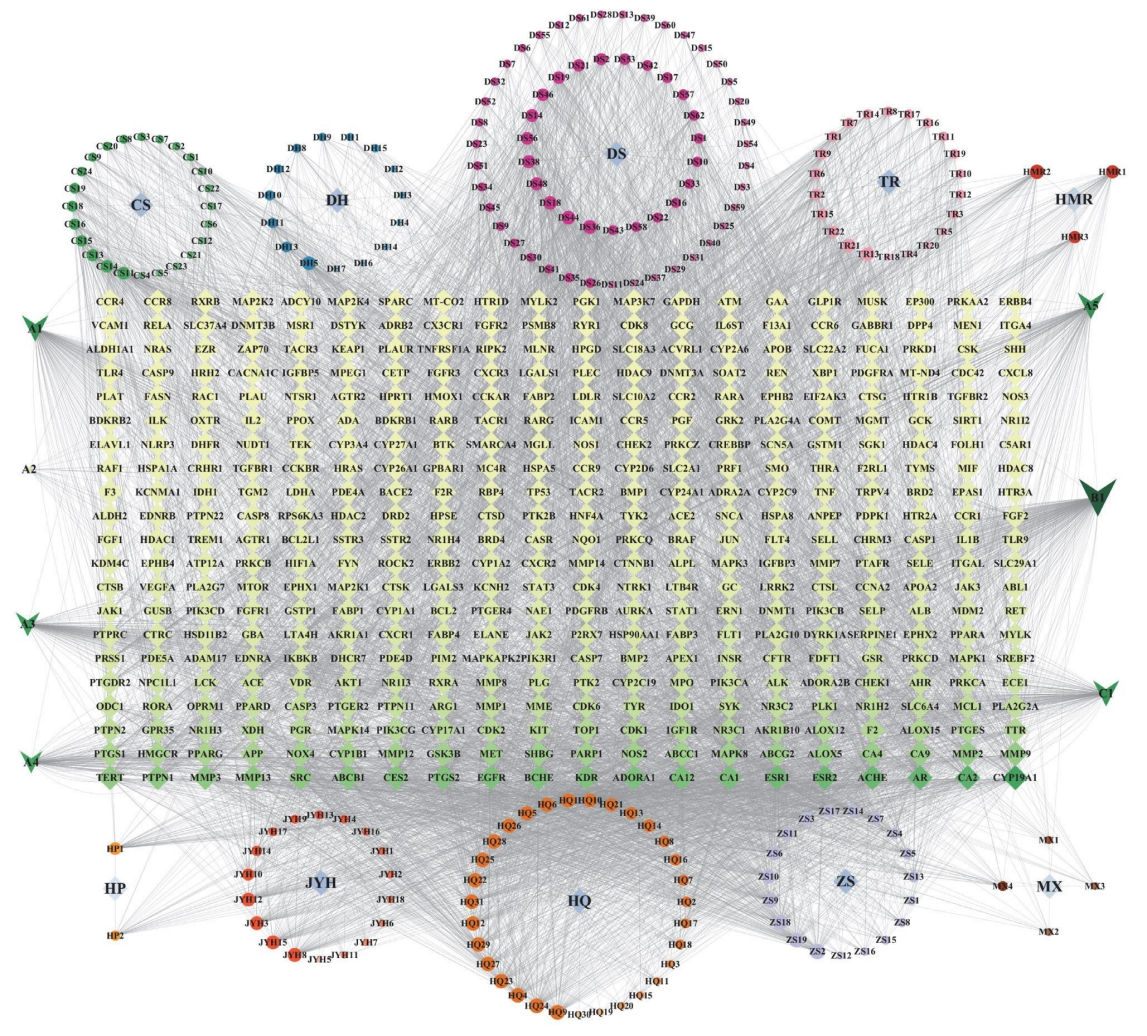
(B)

**Figure:**
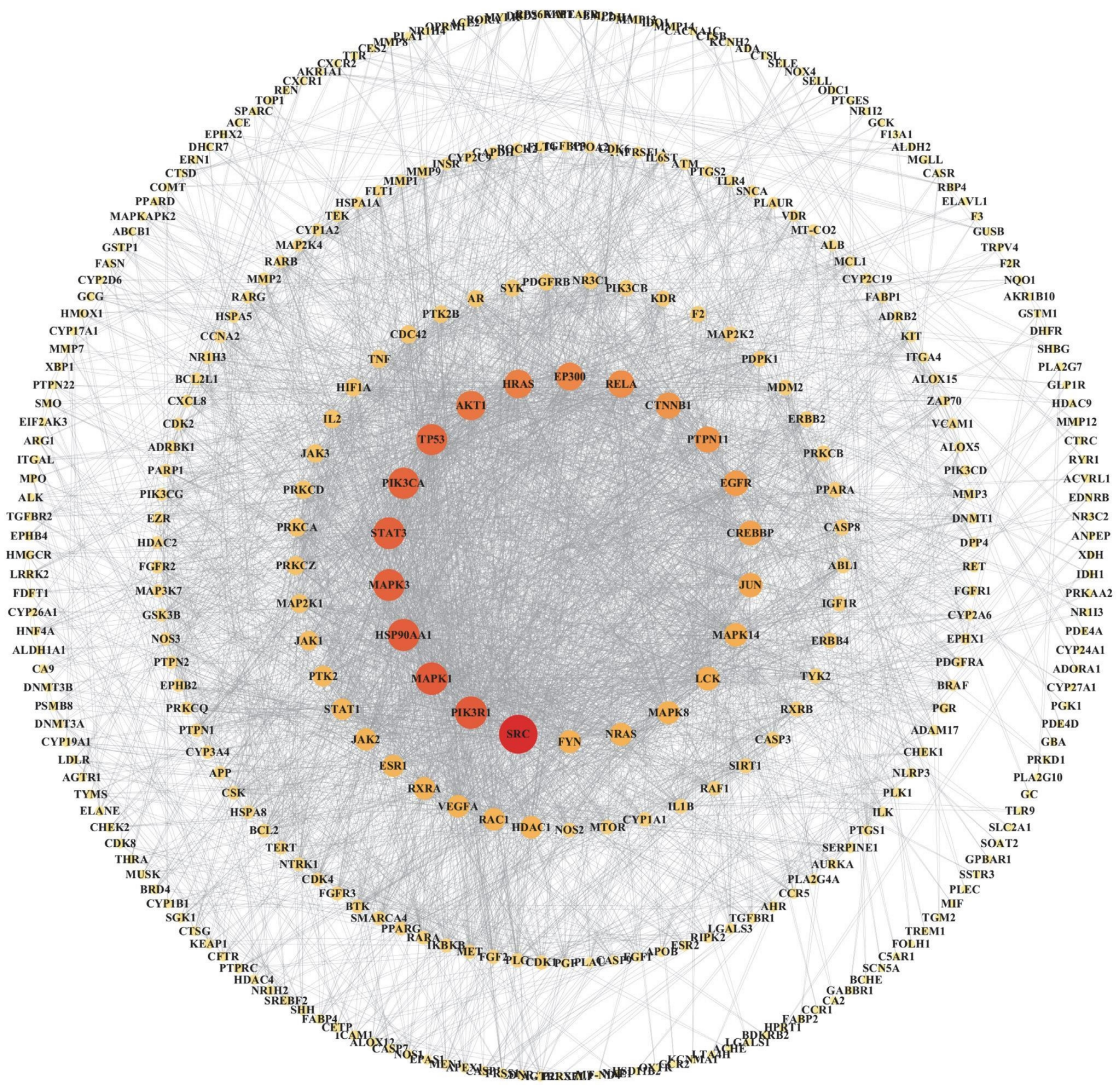
(C)

**Table 1:** Target information of intestinal obstruction.

DisGeNET database	GeneCards database	Total (remove duplicates)
87	2432	2461

Based on this, a “compound‐target‐disease” network was constructed (Figure 2B). The results showed that most compounds acted on multiple targets, with CYP19A1 (aromatase), CA2 (carbonic anhydrase 2), AR (androgen receptor), AChE (acetylcholinesterase), and ESR2 (estrogen receptor β) identified as the top shared nodes. These targets are mainly involved in hormone metabolism, acid‐base and ion homeostasis, immune regulation, neural signaling, and estrogen‐mediated pathways, respectively. The related active compounds are listed in Table 2, with abbreviations shown in Table 3.

**Table 2:** High correlation active ingredients (top 10).

MOL	Molecule name	Source
MOL000006	Luteolin	DS, HMR, JYH, ZS
MOL000449	Stigmasterol	CS, HQ, HMR, JYH, MX
MOL000358	Beta‐sitosterol	CS, DH, HQ, JYH, TR
MOL002914	Eriodyctiol (flavanone)	HQ, JYH, ZS
MOL002714	Baicalein	CS, HQ
MOL000359	Sitosterol	CS, HQ, HMR, MX
MOL013279	5,7,4′‐Trimethylapigenin	ZS
MOL002933	5,7,4′‐Trihydroxy‐8‐methoxyflavone	HQ
MOL000525	Norwogonin	HQ
MOL002927	Skullcapflavone II	HQ

**Table 3:** Ingredient abbreviation table.

Abbreviation	MOL ID	Source	Abbreviation	MOL ID	Source
CS1	MOL007025	CS	HQ2	MOL002911	HQ
CS2	MOL007022	CS	HQ3	MOL002917	HQ
CS3	MOL007018	CS	HQ4	MOL000552	HQ
CS4	MOL007014	CS	HQ5	MOL002909	HQ
CS5	MOL007005	CS	HQ6	MOL002925	HQ
CS6	MOL006996	CS	HQ7	MOL012245	HQ
CS7	MOL006994	CS	HQ8	MOL012246	HQ
CS8	MOL006990	CS	HQ9	MOL002933	HQ
CS9	MOL000492	CS	HQ10	MOL002908	HQ
CS10	MOL007003	CS	HQ11	MOL010415	HQ
CS11	MOL007012	CS	HQ12	MOL001689	HQ
CS12	MOL007008	CS	HQ13	MOL001490	HQ
CS13	MOL006992	CS	HQ14	MOL002910	HQ
CS14	MOL007004	CS	HQ15	MOL001458	HQ
CS15	MOL002883	CS	HQ16	MOL002913	HQ
CS16	MOL006999	CS	HQ17	MOL002926	HQ
CS17	MOL005043	CS	HQ18	MOL002937	HQ
CS18	MOL004355	CS	HQ19	MOL002879	HQ
CS19	MOL001002	CS	HQ20	MOL000073	HQ
CS20	MOL001921	CS	HQ21	MOL002897	HQ
CS21	MOL001925	CS	HQ22	MOL008206	HQ
CS22	MOL001924	CS	HQ23	MOL002934	HQ
CS23	MOL001918	CS	HQ24	MOL000525	HQ
CS24	MOL007016	CS	HQ25	MOL002928	HQ
DH1	MOL000096	DH	HQ26	MOL002932	HQ
DH2	MOL000471	DH	HQ27	MOL012266	HQ
DH3	MOL002297	DH	HQ28	MOL002915	HQ
DH4	MOL002288	DH	HQ29	MOL002927	HQ
DH5	MOL002235	DH	HQ30	MOL001506	HQ
DH6	MOL000554	DH	HQ31	MOL000173	HQ
DH7	MOL002251	DH	HMR1	MOL000483	HMR
DH8	MOL002303	DH	HMR2	MOL001439	HMR
DH9	MOL002259	DH	HMR3	MOL005030	HMR
DH10	MOL002260	DH	JYH1	MOL003006	JYH
DH11	MOL002268	DH	JYH2	MOL003062	JYH
DH12	MOL002293	DH	JYH3	MOL003095	JYH
DH13	MOL002276	DH	JYH4	MOL003101	JYH
DH14	MOL002280	DH	JYH5	MOL002773	JYH
DH15	MOL002281	DH	JYH6	MOL003108	JYH
DS1	MOL007132	DS	JYH7	MOL003111	JYH
DS2	MOL007155	DS	JYH8	MOL003044	JYH
DS3	MOL007150	DS	JYH9	MOL003128	JYH
DS4	MOL007070	DS	JYH10	MOL001495	JYH
DS5	MOL007048	DS	JYH11	MOL003117	JYH
DS6	MOL007140	DS	JYH12	MOL000422	JYH
DS7	MOL001601	DS	JYH13	MOL003059	JYH
DS8	MOL007127	DS	JYH14	MOL001494	JYH
DS9	MOL007050	DS	JYH15	MOL000098	JYH
DS10	MOL007041	DS	JYH16	MOL003014	JYH
DS11	MOL007059	DS	JYH17	MOL003124	JYH
DS12	MOL007045	DS	JYH18	MOL003036	JYH
DS13	MOL007049	DS	MX1	MOL010813	MX
DS14	MOL007036	DS	MX2	MOL010828	MX
DS15	MOL007051	DS	MX3	MOL010839	MX
DS16	MOL007107	DS	MX4	MOL000211	MX
DS17	MOL007088	DS	TR1	MOL001328	TR
DS18	MOL007082	DS	TR2	MOL001329	TR
DS19	MOL007081	DS	TR3	MOL001368	TR
DS20	MOL007094	DS	TR4	MOL001349	TR
DS21	MOL007093	DS	TR5	MOL000493	TR
DS22	MOL002651	DS	TR6	MOL001350	TR
DS23	MOL007098	DS	TR7	MOL001352	TR
DS24	MOL000569	DS	TR8	MOL001353	TR
DS25	MOL007100	DS	TR9	MOL001355	TR
DS26	MOL007101	DS	TR10	MOL001360	TR
DS27	MOL007105	DS	TR11	MOL001361	TR
DS28	MOL007058	DS	TR12	MOL001339	TR
DS29	MOL007108	DS	TR13	MOL001340	TR
DS30	MOL001942	DS	TR14	MOL001342	TR
DS31	MOL007111	DS	TR15	MOL001343	TR
DS32	MOL007115	DS	TR16	MOL001344	TR
DS33	MOL007061	DS	TR17	MOL001358	TR
DS34	MOL007118	DS	TR18	MOL001348	TR
DS35	MOL007119	DS	TR19	MOL001351	TR
DS36	MOL007120	DS	TR20	MOL000296	TR
DS37	MOL007121	DS	TR21	MOL001371	TR
DS38	MOL007123	DS	TR22	MOL001323	TR
DS39	MOL007122	DS	ZS1	MOL009053	ZS
DS40	MOL007124	DS	ZS2	MOL013279	ZS
DS41	MOL007125	DS	ZS3	MOL005100	ZS
DS42	MOL007149	DS	ZS4	MOL013437	ZS
DS43	MOL001771	DS	ZS5	MOL001941	ZS
DS44	MOL001659	DS	ZS6	MOL005849	ZS
DS45	MOL007130	DS	ZS7	MOL013436	ZS
DS46	MOL007064	DS	ZS8	MOL013428	ZS
DS47	MOL007068	DS	ZS9	MOL013277	ZS
DS48	MOL007069	DS	ZS10	MOL004328	ZS
DS49	MOL007152	DS	ZS11	MOL001798	ZS
DS50	MOL007071	DS	ZS12	MOL005828	ZS
DS51	MOL007141	DS	ZS13	MOL013352	ZS
DS52	MOL007142	DS	ZS14	MOL013435	ZS
DS53	MOL007143	DS	ZS15	MOL013276	ZS
DS54	MOL007085	DS	ZS16	MOL013433	ZS
DS55	MOL007145	DS	ZS17	MOL013430	ZS
DS56	MOL007077	DS	ZS18	MOL001803	ZS
DS57	MOL002222	DS	ZS19	MOL007879	ZS
DS58	MOL007079	DS	A1	MOL000358	CS, DH, HQ, JYH, TR
DS59	MOL007151	DS	A2	MOL002776	CS, DS
DS60	MOL007156	DS	A3	MOL002714	CS, HQ
DS61	MOL007154	DS	A4	MOL000359	CS, HQ, HMR, MX
DS62	MOL006824	DS	A5	MOL000449	CS, HQ, HMR, JYH, MX
HP1	MOL005980	HP	B1	MOL000006	DS, HMR, JYH, ZS
HP2	MOL005970	HP	C1	MOL002914	HQ, JYH, ZS
HQ1	MOL000228	HQ	—	—	—

Further PPI network analysis (Figure 2C) included 420 nodes and 2044 edges. According to Degree values, the core targets were SRC (tyrosine kinase Src), PIK3R1 (phosphoinositide‐3‐kinase regulatory subunit 1), MAPK1 (mitogen‐activated protein kinase 1), HSP90AA1 (heat shock protein 90 α family class A member 1), and MAPK3 (mitogen‐activated protein kinase 3). These genes are primarily associated with inflammatory signaling, immune regulation, stress responses, and tissue repair, particularly enriched in the PI3K‐Akt and MAPK/ERK pathways.

In summary, ZWCD may exert therapeutic effects on intestinal obstruction through multicomponent and multitarget synergistic regulation. Notably, CYP19A1, CA2, AR, AChE, and ESR2, along with SRC, PIK3R1, MAPK1/3, and HSP90AA1, play important roles in inflammation regulation, barrier repair, and signal transduction.

### 3.3. Component‐Disease Intersecting Target GO Function and KEGG Pathway Enrichment Analysis

GO enrichment analysis identified 3675 significant entries (*p* < 0.01), including 3128 BP, 189 CC, and 358 MF terms. The top 20 entries in each category indicated that the intersecting targets were mainly enriched in cellular components such as the plasma membrane and receptor complexes, as well as biological processes such as protein phosphorylation and response to LPSs (Figure 3A). KEGG pathway enrichment analysis identified 227 significant pathways (*p* < 0.01), with the top 20 most significant pathways shown in Figure 3B and Table 4. Further pathway‐target network analysis (Figure 3C) demonstrated that the intersecting targets were primarily enriched in key pathways, including cancer (hsa05200), lipid and atherosclerosis (hsa05417), the PI3K‐Akt signaling pathway (hsa04151), and the MAPK signaling pathway (hsa04010). Among these, nodes such as SRC, PIK3R1, MAPK1, and MAPK3 showed higher centrality within the network.

Figure 3GO function and KEGG pathway enrichment analysis of component‐disease intersecting targets. (A) GO enrichment analysis of intersecting targets in the BP, CC, and MF categories; the top 20 terms in each category are shown. (B) KEGG enrichment analysis showing the top 20 pathways. Node size indicates the number of enriched genes, and color represents statistical significance. (C) Pathway‐target network constructed using Cytoscape, where circular nodes represent core pathways and rectangular nodes represent core targets. Node size and color depth correspond to the Degree value.

**Figure:**
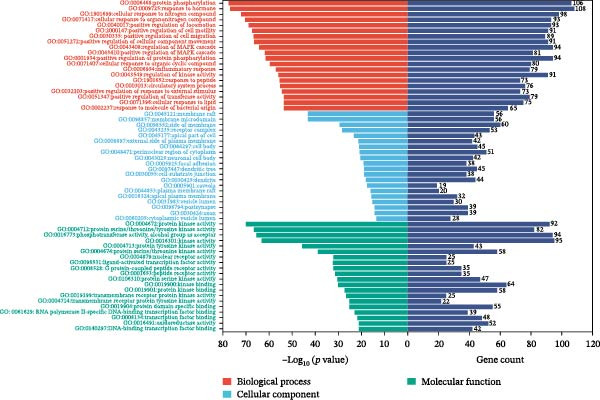
(A)

**Figure:**
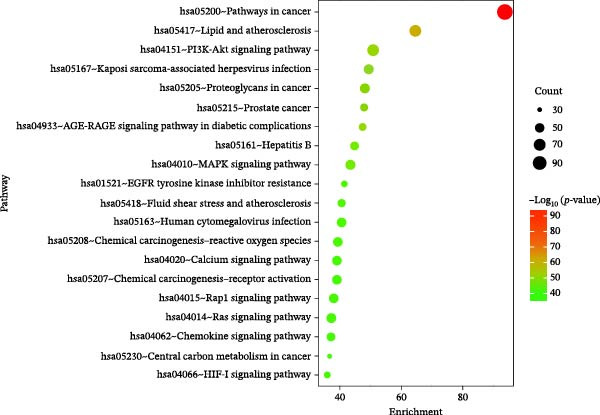
(B)

**Figure:**
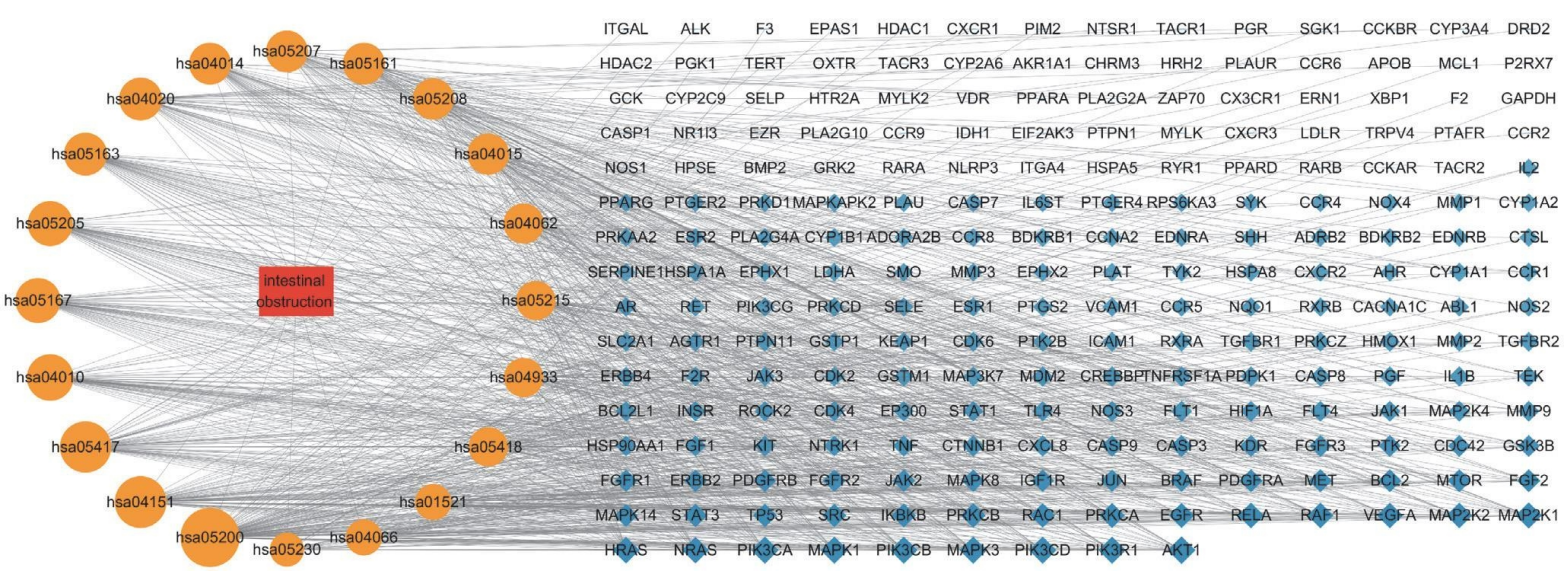
(C)

**Table 4:** KEGG signaling pathways of components‐disease targets (top 20 items).

Pathways	Enrichment	*p* value	Gene count
hsa05200~Pathways in cancer	93.8087	1.55 ^∗^10^−94^	108
hsa05417~Lipid and atherosclerosis	64.5983	2.52 ^∗^10^−65^	63
hsa04151~PI3K‐Akt signaling pathway	51.0833	8.25 ^∗^10^−52^	64
hsa05167~Kaposi sarcoma‐associated herpesvirus infection	49.4541	3.52 ^∗^10^−50^	51
hsa05205~Proteoglycans in cancer	48.1052	7.85 ^∗^10^−49^	51
hsa05215~Prostate cancer	47.9337	1.16 ^∗^10^−48^	40
hsa04933~AGE‐RAGE signaling pathway in diabetic complications	47.2754	5.30 ^∗^10^−48^	40
hsa05161~Hepatitis B	44.8102	1.55 ^∗^10^−45^	45
hsa04010~MAPK signaling pathway	43.3717	4.25 ^∗^10^−44^	54
hsa01521~EGFR tyrosine kinase inhibitor resistance	41.5578	2.77 ^∗^10^−42^	34
hsa05418~Fluid shear stress and atherosclerosis	40.5222	3.00 ^∗^10^−41^	40
hsa05163~Human cytomegalovirus infection	40.4640	3.44 ^∗^10^−41^	47
hsa05208~Chemical carcinogenesis–reactive oxygen species	39.3342	4.63 ^∗^10^−40^	46
hsa04020~Calcium signaling pathway	39.0755	8.40 ^∗^10^−40^	47
hsa05207~Chemical carcinogenesis–receptor activation	39.0700	8.52 ^∗^10^−40^	45
hsa04015~Rap1 signaling pathway	37.9370	1.16 ^∗^10^−38^	44
hsa04014~Ras signaling pathway	37.2125	6.13 ^∗^10^−38^	45
hsa04062~Chemokine signaling pathway	37.0498	8.92 ^∗^10^−38^	42
hsa05230~Central carbon metableolism in cancer	36.6320	2.33 ^∗^10^−37^	30
hsa04066~HIF‐1 signaling pathway	35.8299	1.48 ^∗^10^−36^	34

### 3.4. Molecular Docking Analysis and Mechanism Prediction of Key Components in ZW

**Figure 4:**
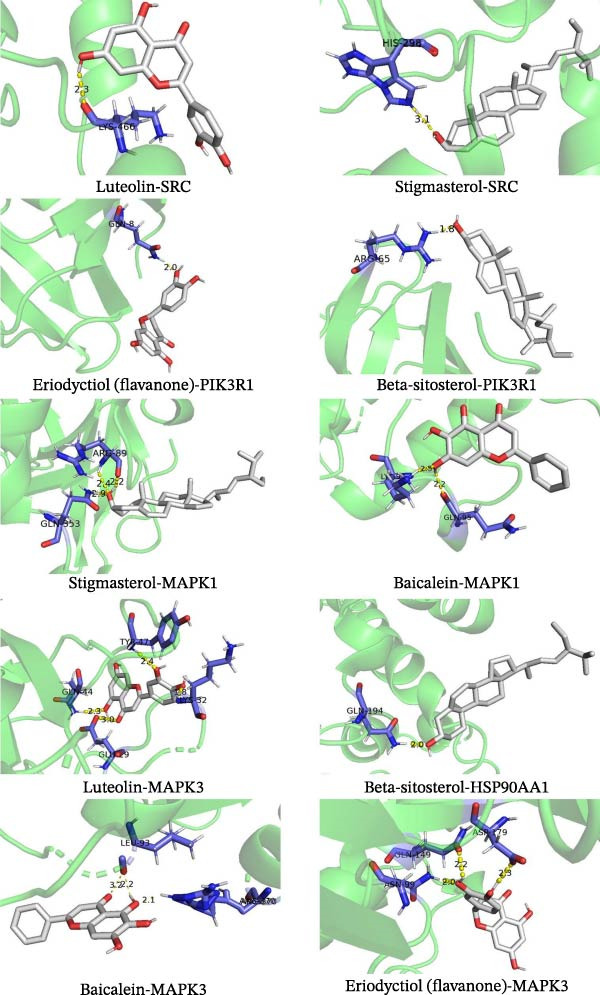
Schematic diagram of some molecular docking results.

To elucidate the key active components and targets of ZWCD, molecular docking was performed between five major compounds (luteolin, stigmasterol, beta‐sitosterol, eriodyctiol, and baicalein), the known MAPK inhibitor SB203580, and five core protein targets (SRC, PIK3R1, MAPK1, HSP90AA1, MAPK3), resulting in 25 docking tests (Figure 4, Table 5). Binding energies ≤−1.2 kcal/mol were considered indicative of strong interactions [29].

**Table 5:** Molecules docking results of key compounds and key targets.

Ligand	Macromolecule	PDB ID	Binding energy (kcal/mol)
Luteolin	SRC	5uut	−1.89
Luteolin	PIK3R1	3o5z	−2.63
Luteolin	MAPK1	2ojg	−4.23
Luteolin	HSP90AA1	6gpo	−2.13
Luteolin	MAPK3	4qtb	−3.61
Stigmasterol	SRC	5uut	−2.89
Stigmasterol	PIK3R1	3o5z	−3.54
Stigmasterol	MAPK1	2ojg	−4.92
Stigmasterol	HSP90AA1	6gpo	−2.74
Stigmasterol	MAPK3	4qtb	−4.00
Beta‐sitosterol	SRC	5uut	−2.16
Beta‐sitosterol	PIK3R1	3o5z	−3.13
Beta‐sitosterol	MAPK1	2ojg	−4.97
Beta‐sitosterol	HSP90AA1	6gpo	−2.17
Beta‐sitosterol	MAPK3	4qtb	−3.66
Eriodyctiol (flavanone)	SRC	5uut	−1.94
Eriodyctiol (flavanone)	PIK3R1	3o5z	−2.26
Eriodyctiol (flavanone)	MAPK1	2ojg	−2.19
Eriodyctiol (flavanone)	HSP90AA1	6gpo	−1.93
Eriodyctiol (flavanone)	MAPK3	4qtb	−2.56
Baicalein	SRC	5uut	−1.52
Baicalein	PIK3R1	3o5z	−2.53
Baicalein	MAPK1	2ojg	−3.03
Baicalein	HSP90AA1	6gpo	−2.51
Baicalein	MAPK3	4qtb	−3.50

Results showed MAPK1 and MAPK3 as the most critical targets: MAPK1 displayed strong affinity with Beta‐sitosterol (−4.97 kcal/mol), Stigmasterol (−4.92 kcal/mol), and Luteolin (−4.23 kcal/mol); MAPK3 showed strong binding with Stigmasterol (−4.00 kcal/mol) and Luteolin (−3.61 kcal/mol). Notably, the binding energies of Luteolin (−4.23, −3.61 kcal/mol) and Stigmasterol (−4.92, −4.00 kcal/mol) to MAPK1/MAPK3 were comparable to or even better than SB203580, suggesting their potential as natural MAPK regulators. Stigmasterol also exhibited strong affinity with PIK3R1 (−3.54 kcal/mol), implying possible involvement in the PI3K‐Akt pathway. In contrast, HSP90AA1 showed relatively weaker binding (−1.93 to −2.74 kcal/mol), indicating it may play a secondary role. Binding mode analysis revealed that these compounds mainly formed stable complexes via hydrogen bonding, hydrophobic interactions, and π–π stacking.

Integrating docking with GO and KEGG enrichment results, Beta‐sitosterol, Stigmasterol, and Luteolin were identified as the core active compounds of ZWCD, with MAPK1 and MAPK3 as major targets. These compounds may regulate inflammatory responses, oxidative stress, and cell survival through MAPK‐related pathways. Literature also supports these findings: Luteolin is reported to have clear anti‐inflammatory and MAPK‐modulating effects [30–32], while Beta‐sitosterol and Stigmasterol exhibit anti‐inflammatory and cardioprotective activities [14, 26, 33, 34], though their roles in intestinal obstruction and MAPK1 regulation remain less studied.

In conclusion, Beta‐sitosterol, Stigmasterol, and Luteolin were identified as key bioactive constituents of ZWCD, with MAPK1 and MAPK3 as primary molecular targets. These findings provide mechanistic insights into the anti‐inflammatory and intestinal repair effects of ZWCD and a theoretical basis for further validation.

### 3.5. Luteolin, Stigmasterol, and Beta‐Sitosterol Alleviate Inflammatory Response in Intestinal Obstruction by Inhibiting the MAPK1/MAPK3 Signaling Pathway

In the 48 h observation period, no mortality occurred in any group of mice, indicating that the modeling and treatment procedures were well tolerated. In this study, the LPS group showed significantly elevated levels of p‐MAPK1/p‐MAPK3, p‐AKT, and PI3K, suggesting activation of the MAPK and PI3K‐Akt signaling pathways. In contrast, Western blot results demonstrated that these protein levels were markedly reduced in the LPS + Luteolin, LPS + Stigmasterol, LPS + β‐sitosterol, and LPS + Octreotide groups (Figure 5A). qPCR analysis further showed downregulation of MAPK1 and MAPK3 mRNA expression (Figure 5B, C).

Figure 5Anti‐inflammatory and intestinal tissue repair effects of Luteolin, Stigmasterol, and Beta‐sitosterol in LPS‐induced inflammation model. (A) Western blot analysis of p‐MAPK1/p‐MAPK3, p‐AKT, and PI3K protein levels in mouse intestinal tissues (*n* = 5). (B, C) qPCR analysis of MAPK1 and MAPK3 mRNA expression (*n* = 5). (D) ELISA analysis of serum IL‐6, TNF‐α, and IL‐1β levels (*n* = 3). (E) H&E staining of ileocecal tissues, scale bar = 100 μm (*n* = 5). Data are presented as mean ± SD,  ^∗^ *p* < 0.05 vs. LPS,  ^#^ *p* < 0.05 vs. Control.

**Figure:**
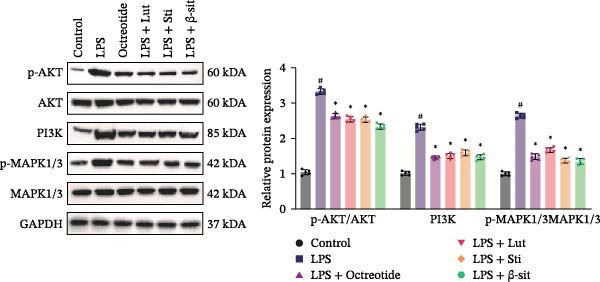
(A)

**Figure:**
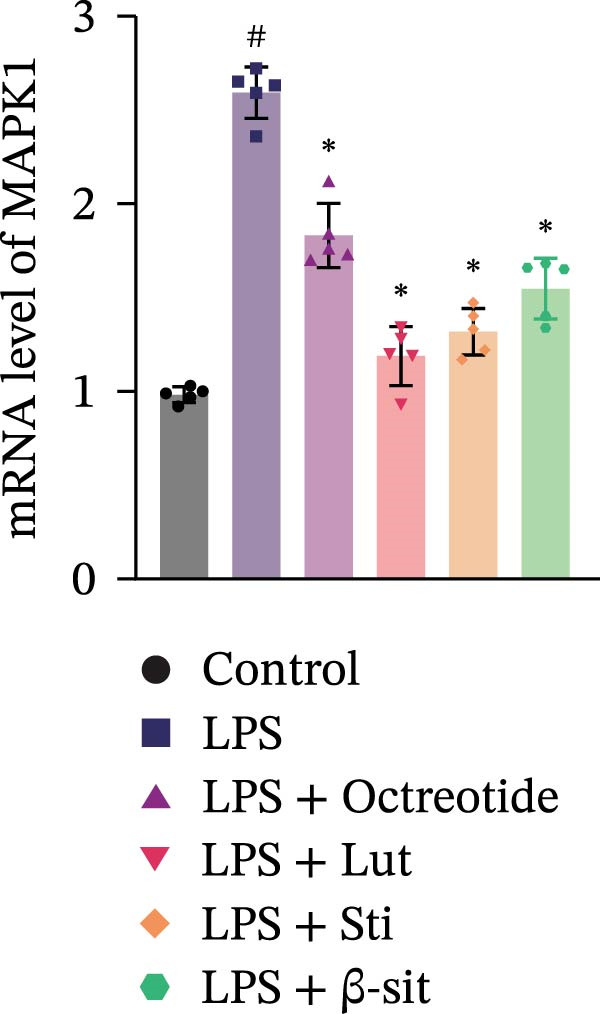
(B)

**Figure:**
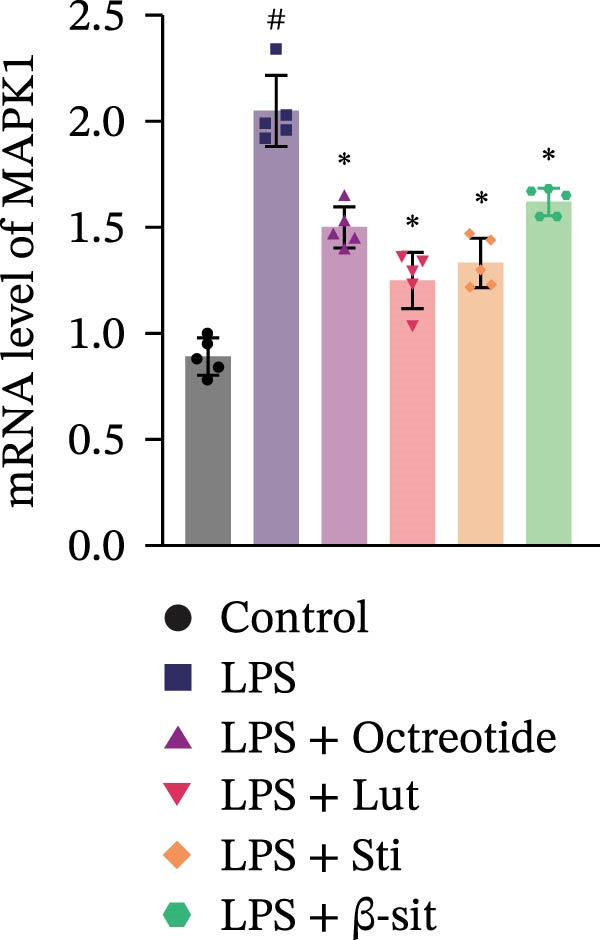
(C)

**Figure:**
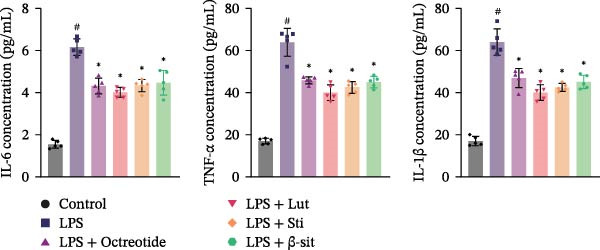
(D)

**Figure:**
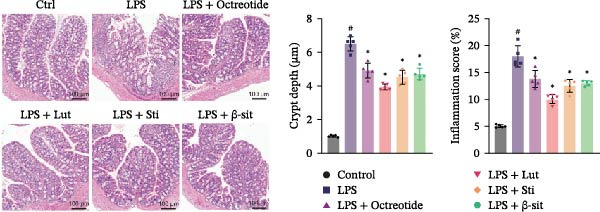
(E)

Serum ELISA results revealed that IL‐6, TNF‐α, and IL‐1β levels were significantly decreased in the LPS + Luteolin, LPS + Stigmasterol, LPS + β‐sitosterol, and LPS + Octreotide groups compared with the LPS group (*p* < 0.01) (Figure 5D), with the most pronounced effect observed in the LPS + Luteolin group. Histological analysis indicated severe ileal injury in the LPS group, characterized by deepened crypts and marked inflammatory cell infiltration. In contrast, treatment with Luteolin, Stigmasterol, β‐sitosterol, or Octreotide significantly alleviated tissue damage, with the LPS + Luteolin group showing the most prominent repair effect (Figure 5E). Taken together, these results suggest that Luteolin, Stigmasterol, and β‐sitosterol may serve as key active components of ZW, exerting anti‐inflammatory and intestinal protective effects primarily through inhibition of the MAPK1/MAPK3 signaling pathway, with Luteolin displaying the strongest therapeutic potential.

### 3.6. The Therapeutic Effects of ZW on Intestinal Obstruction Rats Include Intestinal Histological Repair and Regulation of Inflammatory Factor Levels

Histopathological analysis (Figure 6A–H) showed that the ileal mucosa of the Control group was intact, with well‐organized and clearly defined crypts (Figure 6A), and the Sham‐operated group displayed no significant difference compared with the Control group (Figure 6B). In contrast, the Model group exhibited severe tissue injury, characterized by marked edema and congestion in the serosal layer, extensive infiltration of red blood cells and inflammatory cells, and distorted or branched crypt structures (Figure 6C, D). The Low‐dose ZWCD group still showed evident pathological damage, including serosal edema, crypt loss, and red blood cell aggregation, although the extent of inflammatory cell infiltration was reduced compared with the Model group (Figure 6F). The Medium‐dose ZWCD group exhibited relatively preserved tissue structure and alleviated inflammatory manifestations, with only mild edema and limited inflammatory cell infiltration (Figure 6G). Notably, the Octreotide group and High‐dose ZWCD group demonstrated the most significant improvement, with intact ileal structure, well‐defined crypt boundaries, markedly reduced congestion and red blood cell infiltration, and only mild edema in a few regions (Figure 6E, H).

Figure 6Pathological effects of Zengwei Chengqi Decoction on cecal tissue in intestinal obstruction rats. (A) Control group (*n* = 5), no surgical intervention. (B) Sham‐operated group (*n* = 5), mesenteric puncture without intestinal ligation. (C, D) Model group (*n* = 5), BaCl_2_‐induced intestinal obstruction without treatment. (E) Octreotide group (*n* = 5), octreotide 50 μg/kg. (F) Low‐dose ZWCD group (*n* = 5), 5 mL/(kg d). (G) Medium‐dose ZWCD group (*n* = 5), 10 mL/(kg d). (H) High‐dose ZWCD group (*n* = 5), 20 mL/(kg d), representative H&E‐stained sections are shown in (A–H), scale bar = 100 μm. (I) Serum IL‐1β, IL‐6, and TNF‐α levels by ELISA, data are presented as Mean ± SD, with  ^∗∗^ *p* < 0.01. (J) Ultrastructural changes in colonic tissues under TEM, scale bar = 1 μm.

**Figure:**
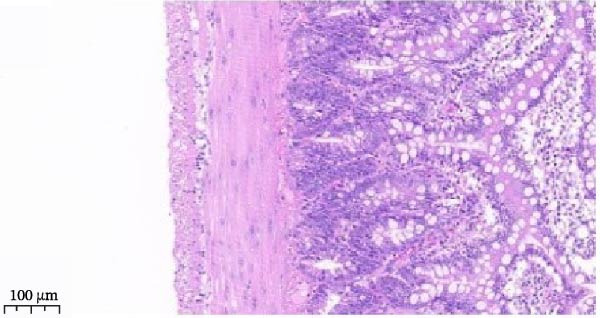
(A)

**Figure:**
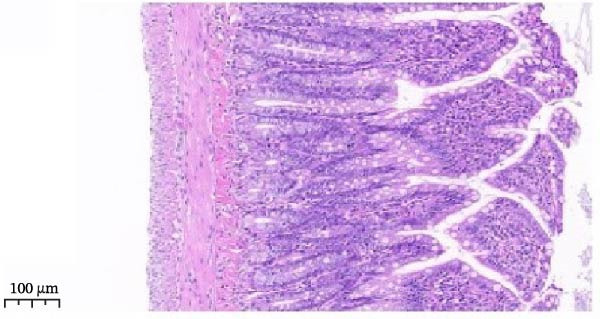
(B)

**Figure:**
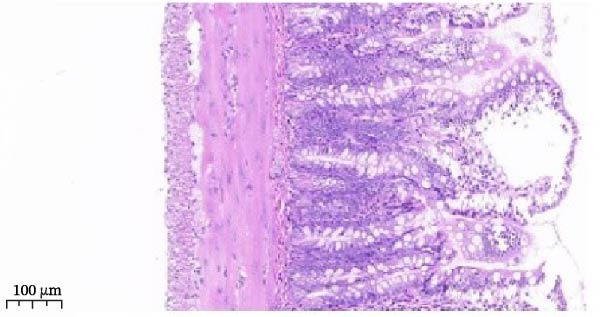
(C)

**Figure:**
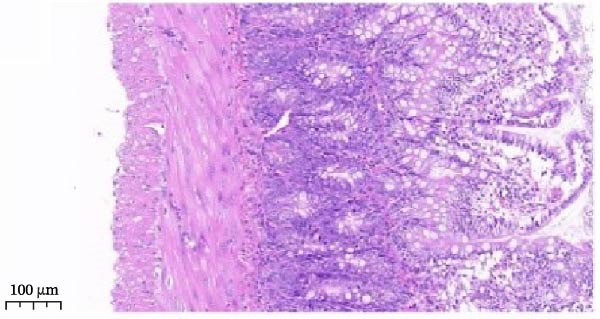
(D)

**Figure:**
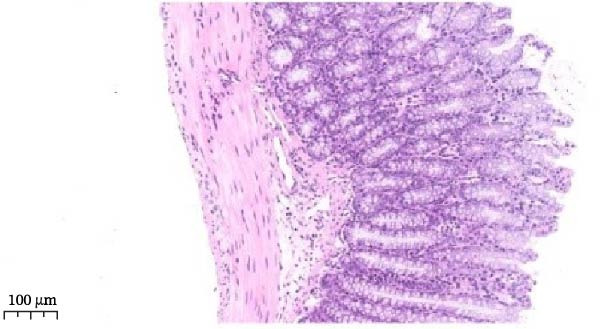
(E)

**Figure:**
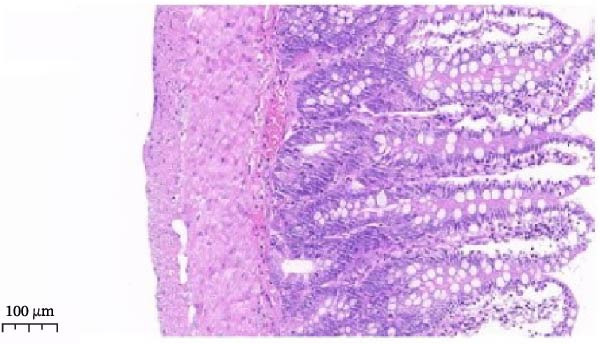
(F)

**Figure:**
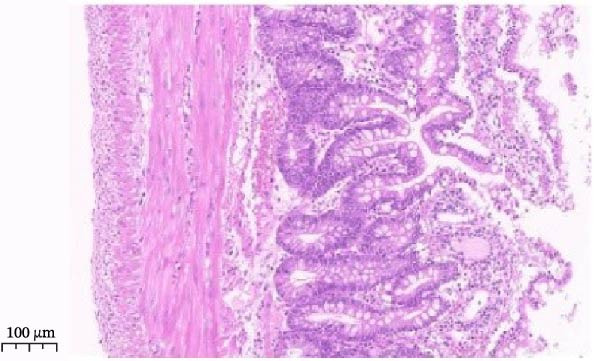
(G)

**Figure:**
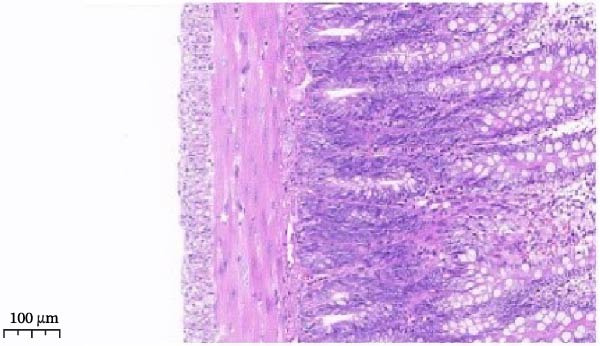
(H)

**Figure:**
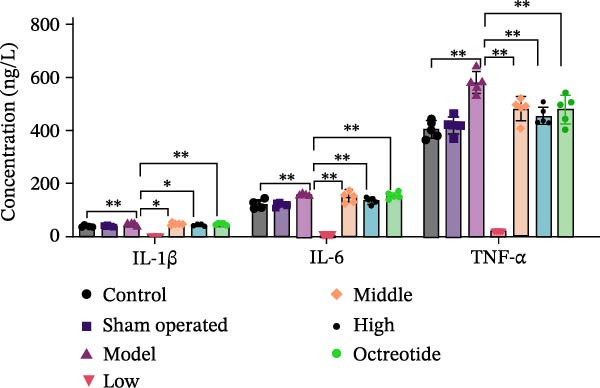
(I)

**Figure:**
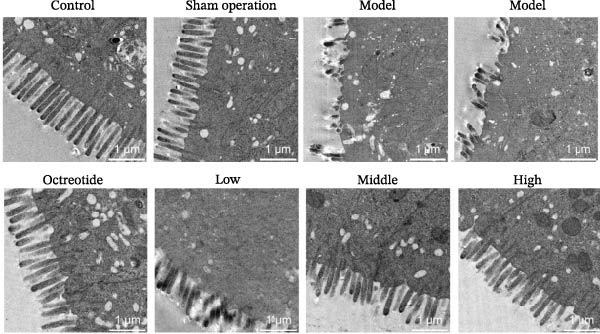
(J)

TEM further revealed that the colonic ultrastructure of the Control group showed normal microvilli morphology and intact, narrow tight junctions (Figure 6J). In contrast, the Model group displayed severe TJ disruption, cystic dilatation of intercellular spaces, and extensive microvilli sparsity or loss. All treatment groups exhibited varying degrees of improvement, with the High‐dose ZWCD group showing the best preservation of microvilli morphology and TJ integrity, along with tightly arranged cell membranes. The Octreotide group also showed marked ultrastructural recovery.

ELISA results (Figure 6I, Table 6) indicated that serum IL‐1β, IL‐6, and TNF‐α levels were significantly elevated in the Model group compared with the Control group (*p* < 0.01), while no significant differences were observed between the Sham‐operated and Control groups (*p* > 0.05). Both the Low‐dose and Medium‐dose ZWCD groups showed partial reductions in inflammatory cytokines, with IL‐1β decreased compared with the Model group (*p* < 0.05) and IL‐6 and TNF‐α significantly reduced (*p* < 0.01). The High‐dose ZWCD group exhibited the most pronounced anti‐inflammatory effect, with IL‐1β, IL‐6, and TNF‐α levels significantly lower than those in the Model group (*p* < 0.01).

**Table 6:** IL‐1β, IL‐6 and TNF‐α level in serum of rats in each group (x̄ ± *s*) (*n* = 6).

Group	IL‐1β (ng/L)	IL‐6 (pg/mL)	TNF‐α (ng/L)
Low	44.24 ± 1.91^a^	147.59 ± 7.45^b^	412.77 ± 22.44^b^
Middle	47.03 ± 2.89^a^	147.18 ± 5.65^b^	481.74 ± 35.39^b^
High	45.24 ± 2.03^b^	134.68 ± 4.20^b^	455.73 ± 24.65^b^
Model	51.03 ± 4.55^d^	159.17 ± 7.09^d^	581.07 ± 51.45^d^
Sham operation	41.77 ± 3.53	122.06 ± 4.14	420.69 ± 50.61
Control	39.59 ± 2.23	119.74 ± 4.28	404.00 ± 51.95
*F*	0.074	0.087	0.065

*Note:* ^a^ indicates *p* < 0.05 compared with the model group, ^b^ indicates *p* < 0.01 compared with the model group, ^d^ Indicates *p* < 0.01 compared with the blank control group.

Taken together, these findings suggest that ZWCD effectively attenuates intestinal pathological injury and ultrastructural damage while reducing systemic inflammation in rats with intestinal obstruction. The therapeutic effects were dose‐dependent, with the High‐dose ZWCD group showing the most significant efficacy, indicating that ZWCD may exert protective effects by modulating inflammatory responses and enhancing intestinal barrier repair.

## 4. Discussion

AIO is a common life‐threatening surgical emergency characterized by partial or complete blockage of the intestinal tract. It can cause abdominal pain, distension, and nausea, and in severe cases, may lead to intestinal ischemia, necrosis, and even death [1, 4, 35–37]. Current clinical treatments mainly rely on surgery and pharmacological interventions. However, surgery carries high risks and trauma, making pharmacological approaches an important complement. TCM has shown unique advantages in the treatment of AIO. Among these, ZWCD has been widely applied to alleviate related symptoms [7, 38, 39]. Nevertheless, its exact mechanism of action remains unclear.

This study combined network pharmacology and animal experiments, demonstrating that ZWCD significantly reduced serum levels of inflammatory cytokines such as IL‐1β, IL‐6, and TNF‐α, while improving pathological damage in rat models of intestinal obstruction. These findings suggest that its therapeutic effects may be achieved through a multicomponent, multitarget mechanism that exerts anti‐inflammatory and protective actions, providing new experimental evidence for clinical applications.

Originating from modifications of Wenbing Tiaobian, ZWCD is characterized by moistening dryness, nourishing yin, regulating qi, and unblocking the bowels, and is often prescribed for severe abdominal pain, distension, and constipation [40]. In terms of herbal composition, rhubarb (Dahuang) serves as the monarch herb with its bitter‐cold properties that clear heat and purge the bowels; mirabilite (Mangxiao) acts as the minister herb by softening hardness and moistening dryness; salvia (Danshen) nourishes yin and protects against damage; magnolia bark (Houpo) and immature bitter orange (Zhishi) regulate qi and relieve stagnation; honeysuckle (Jinyinhua) clears heat and cools blood; and red peony root (Chishao) alleviates pain and spasms. Together, they form a synergistic prescription [41, 42]. The formula embodies the coordination of attacking pathogenic factors while supporting the body and combining purgation with yin protection. Recent studies have confirmed its therapeutic effects: for instance, it improves intestinal dysfunction in septic rats by modulating inflammation and immune responses [23]; it also promotes recovery of gastrointestinal function and wound healing after mixed hemorrhoid surgery when combined with Kushen Decoction fumigation [43]. Compared with previous reports, the present study not only clarified active components and potential targets but also validated their effects in animal models, providing more comprehensive supporting evidence.

Network pharmacology analysis revealed that ZWCD involves 228 active compounds and 996 potential targets, constructing a systematic interaction network. Previous studies suggested that PI3K‐Akt and MAPK pathways play important roles in intestinal obstruction [44], but their interactions remain insufficiently understood. This study uncovered the dual regulatory role of MAPK1/3 (ERK1/2): excessive activation promotes the release of pro‐inflammatory cytokines such as IL‐1β and TNF‐α via AP‐1 phosphorylation, while also disrupting tight junction proteins; in contrast, moderate activation aids mucosal repair and regeneration [45, 46]. In the PI3K‐Akt pathway, the key target PIK3R1 enhances tight junction integrity and promotes mucosal healing, thereby mitigating inflammatory damage [47, 48]. Furthermore, this study confirmed that ZWCD significantly regulates the SRC gene. As a critical signaling molecule, SRC exhibits high connectivity within the PPI network and regulates epithelial cell migration and proliferation via FAK and PI3K pathways, contributing to mucosal barrier repair. This highlights SRC’s therapeutic potential in intestinal obstruction [49]. This discovery expands the focus beyond inflammatory cytokines, suggesting that regulation of SRC may facilitate mucosal repair and alleviate inflammation through its roles in cell proliferation and apoptosis.

Compared with synthetic MAPK inhibitors such as SB203580, which can rapidly block MAPK1/3 overactivation to suppress inflammation, single‐target drugs fail to address multiple pathological processes such as dysmotility and mucosal repair and carry risks of off‐target effects [50, 51]. In contrast, the multicomponent and multitarget nature of ZWCD allows it to regulate MAPK1/3 balance for anti‐inflammatory effects, enhance intestinal barrier function via PI3K‐Akt, promote repair via SRC, and improve motility through herbs like Houpo and Zhishi. This results in a synergistic “anti‐inflammation‐repair‐motility regulation” effect [45, 46, 49], aligning better with the complex pathology of intestinal obstruction and reflecting the integrative, holistic advantages of TCM formulas.

The findings of this study provide scientific support for the clinical use of ZWCD in treating AIO. By combining network pharmacology’s multitarget analysis with experimental validation, the study offers a foundation for more individualized therapeutic strategies, particularly for patients who respond poorly to conventional treatments. The results suggest that ZWCD may be applied alongside western medical interventions to achieve enhanced outcomes through both anti‐inflammatory and mucosal repair mechanisms, with dosage and treatment duration adjustable based on patient‐specific conditions.

Nonetheless, the study has several limitations. First, the functional roles and interactions of identified targets have not been validated at the cellular level, and the pharmacokinetics of active components remain unclear, limiting interpretation of their strength and duration of action. Second, the animal experiments had small sample sizes with differences from clinical conditions, and only short‐term effects were evaluated, restricting generalizability. Third, clinical validation involved only a small number of patients without large‐scale randomized controlled trials, reducing statistical power and feasibility. Fourth, dose‐response and safety profiles across diverse populations remain underexplored, raising concerns about individual variability in treatment outcomes. Fifth, TEM was not applied to assess mucosal ultrastructure, a critical indicator of barrier damage and repair. Finally, the study mainly focused on MAPK and PI3K‐Akt pathways, without exploring other inflammatory signaling such as NF‐κB, which may interact in complex ways. Thus, the multi‐pathway synergistic mechanisms of ZWCD remain insufficiently elucidated.

Future studies may address these gaps by: (1) conducting cellular experiments to validate core targets in inflammatory regulation and signaling activation; (2) using single‐component isolation to determine the contribution of key compounds to overall efficacy; (3) carrying out large‐scale randomized controlled trials to systematically evaluate efficacy and safety; (4) optimizing dosages for different populations to enable personalized treatment; (5) developing novel anti‐inflammatory small molecules or combined therapies based on key compounds and targets; (6) employing nanocarrier drug delivery systems to improve bioavailability, efficacy, and safety; (7) conducting long‐term studies in chronic intestinal obstruction and related inflammatory diseases to assess feasibility as adjunctive or alternative therapy; (8) applying AI and multi‐omics approaches to integrate target modeling and broaden applications to other inflammatory conditions; (9) incorporating TEM to assess effects on intestinal mucosal ultrastructure; (10) systematically analyzing cross‐talk among NF‐κB, MAPK, and PI3K‐Akt pathways for a more comprehensive understanding of the synergistic anti‐inflammatory mechanisms.

## 5. Conclusion

This study systematically elucidates the multicomponent, multitarget, and multi‐pathway mechanisms of ZW in treating intestinal obstruction through network pharmacology and animal experiments. The research finds that the key active components of ZW regulate multiple core targets (such as SRC, PIK3R1, and MAPK1), significantly inhibiting the expression of inflammatory factors (IL‐1β, IL‐6, and TNF‐α) and improving intestinal pathological structural damage. The high‐dose group demonstrates the best therapeutic effect, with a significant reduction in tissue inflammation and the most notable functional recovery. This study not only validates the anti‐inflammatory and repair effects of ZW but also provides new scientific evidence for the use of TCM in treating intestinal obstruction.

**Nomenclature**
AChE: Acetylcholinesterase
ANOVA: Analysis of variance
AR: Androgen receptor
BP: Biological processes
CA2: Carbonic anhydrase 2
CC: Cellular components
CYP19A1: Aromatase
CRP: C‐reactive protein
CS: Chi Shao
DS: Dan Shen
DH: Da Huang
DL: Drug‐likeness
ESR2: Estrogen receptor β
ELISA: Enzyme‐linked immunosorbent assay
FBS: Fetal bovine serum
GO: Gene ontology
H&E: Hematoxylin and eosin
HMR: Huo Ma Ren
HQ: Huang Qin
HP: Hou Po
HSP90AA1: Heat shock protein 90 alpha family class A member 1
hs‐CRP: High‐sensitivity C‐reactive protein
IL‐1β: Interleukin‐1β
IL‐6: Interleukin‐6
JYH: Jin Yin Hua
*KEGG*: Kyoto encyclopedia of genes and genomes
MAPK1: Mitogen‐activated protein kinase 1
MAPK3: Mitogen‐activated protein kinase 3
Mean ± SD: Mean ± standard deviation
MF: Molecular functions
MX: Mu Xiang
MGX: Mang Xiao
OB: Oral bioavailability
OD: Optical density
PPI: Protein–protein interaction
PIK3R1: Phosphoinositide‐3‐kinase regulatory subunit 1
qPCR: Quantitative polymerase chain reaction
TCM: Traditional Chinese medicine
TCMSP: Traditional Chinese medicine systems pharmacology database and analysis platform
TNF‐α: Tumor necrosis factor‐α
TR: Tao Ren
Urinary AMY: Urinary amylase
WBC: White blood cell count
ZS: Zhi Shi.

## Author Contributions

Ying Gong, Zi‐Hua Xuan, Cong‐Bin Liu, and Dong‐Mei Xie conceived and designed the study. Ying Gong, Zi‐Hua Xuan, Yue Dong, Chun‐Bin Zhang, Yu‐Chen Liu, Jin‐Hui Zhang, and Qing Li performed the experiments, analyzed the data. Ying Gong and Zi‐Hua Xuan wrote the manuscript.

## Funding

This study was supported by the Chuzhou Science and Technology Plan Project (Grant 2021ZN006) and Team Project of Anhui University Research Program (Grant 2022AH010036).

## Disclosure

All authors reviewed and approved the final version of the manuscript.

## Ethics Statement

This study was approved by the Clinical Ethics Committee of Anhui University of Chinese Medicine (Number 2024‐2420‐01). All the patients have been informed and signed informed consent before the experiments. All animal experiments were approved by the Animal Ethics Committee of Anhui University of Chinese Medicine (Number 2024010).

## Consent

The authors have nothing to report.

## Conflicts of Interest

The authors declare no conflicts of interest.

## Supporting Information

Additional supporting information can be found online in the Supporting Information section.

## Supporting information


**Supporting Information 1** Figure S1: Schematic diagram of the treatment process for both groups of patients.

**Supporting Information 2** Table S1: Baseline characteristics of the two groups.

**Supporting Information 3** Figure S2: Schematic diagram of experimental animal grouping, modeling, and drug administration.

## Data Availability

The data that support the findings of this study are available upon request from the corresponding author.
