# Supplementary figures and images for: Zengwei Chengqi Decoction Reduces Inflammation in Acute Intestinal Obstruction
Source: Mediators Inflamm. 2026 Mar 13;2026:8826129. doi: 10.1155/mi/8826129 (PMC13140358; doi:10.1155/mi/8826129)

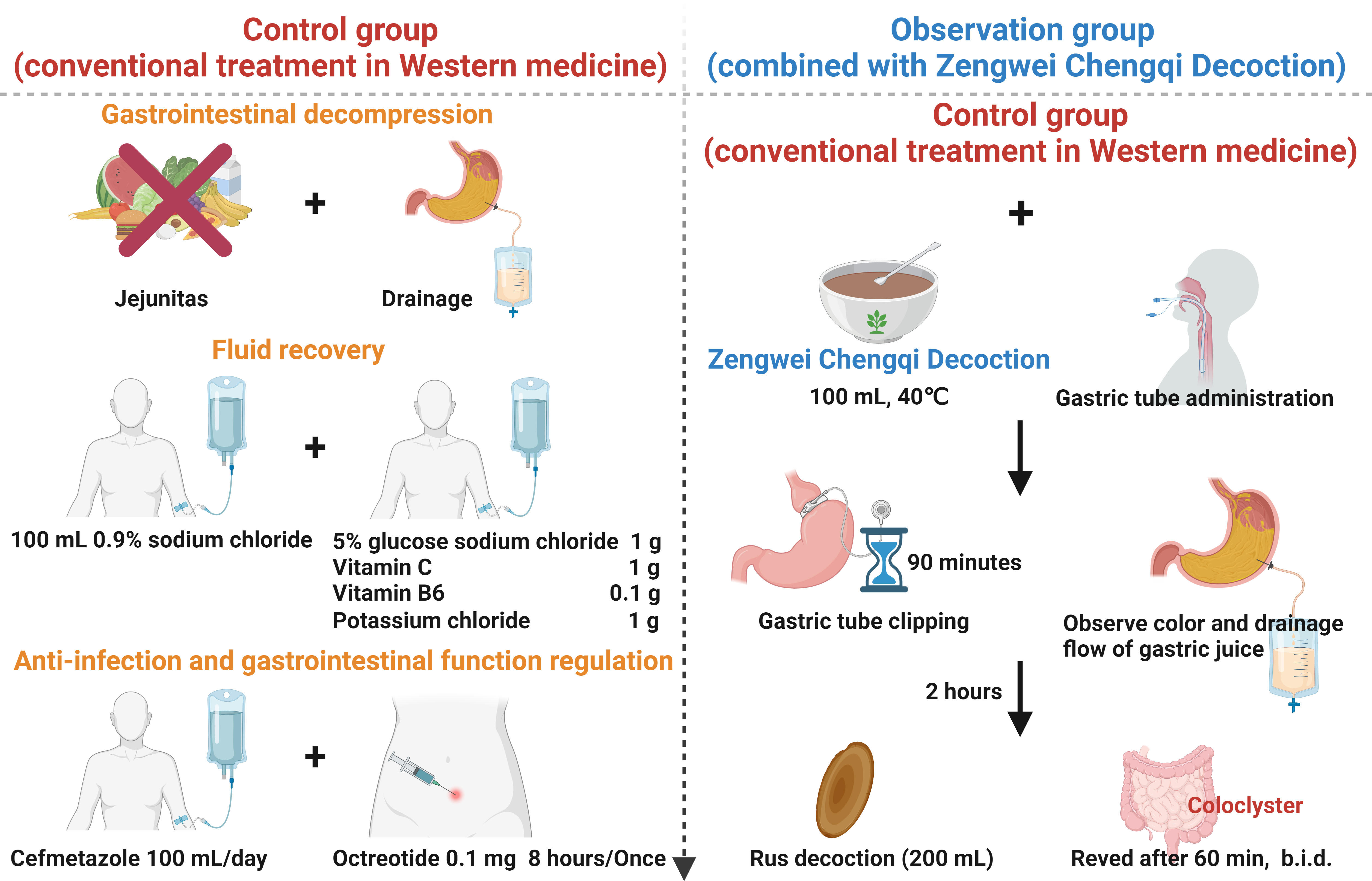

Supplement: Supplementary file 1 — Supporting Information 1 Figure S1: Schematic diagram of the treatment process for both groups of patients. [file MI-2026-8826129-s001.jpeg]

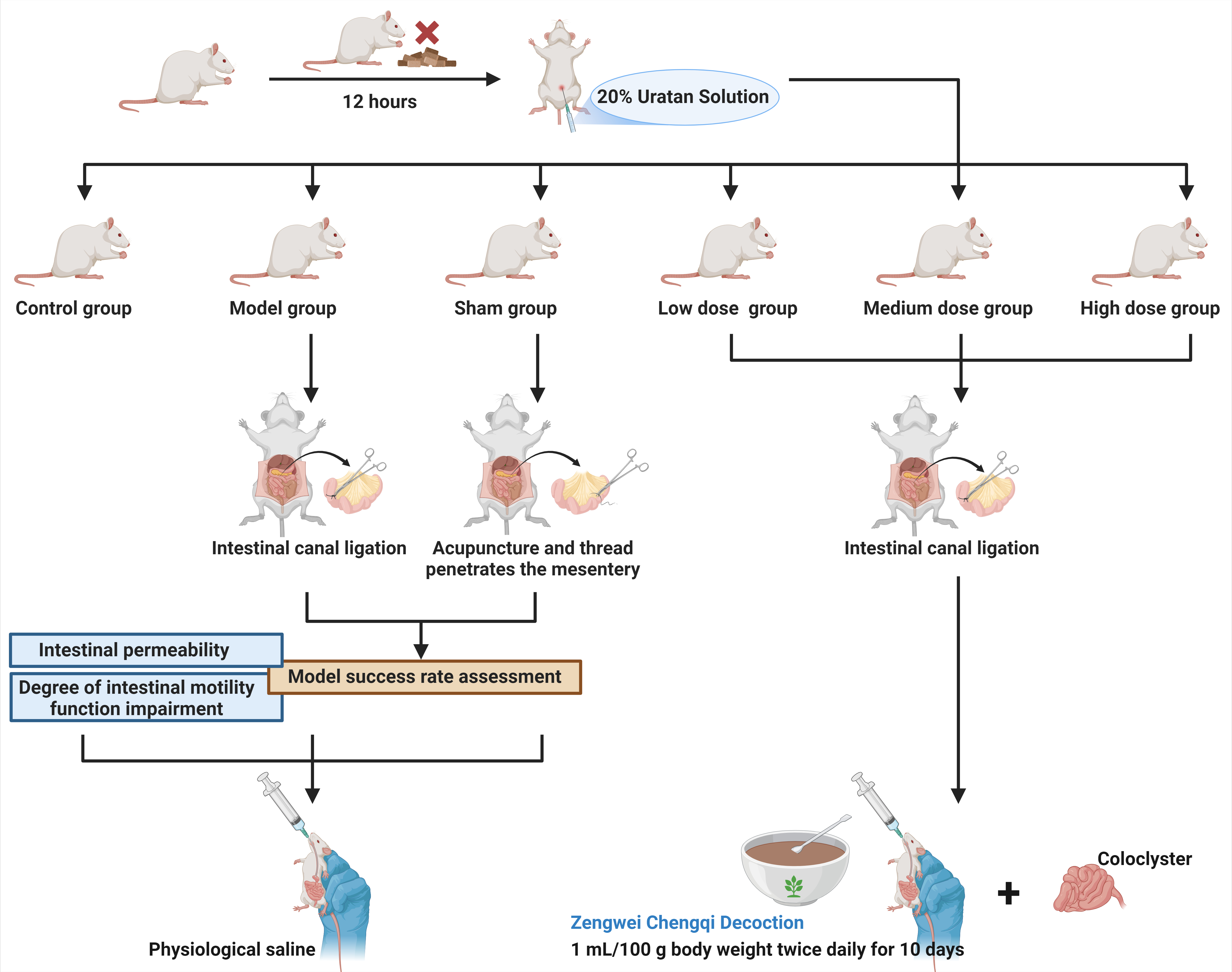

Supplement: Supplementary file 3 — Supporting Information 3 Figure S2: Schematic diagram of experimental animal grouping, modeling, and drug administration. [file MI-2026-8826129-s003.jpeg]
