# Supplementary material for: Zengwei Chengqi Decoction Reduces Inflammation in Acute Intestinal Obstruction
Source: Mediators Inflamm. 2026 Mar 13;2026:8826129. doi: 10.1155/mi/8826129 (PMC13140358; doi:10.1155/mi/8826129)
Supplement: Supplementary file 2 — Supporting Information 2 Table S1: Baseline characteristics of the two groups. [file MI-2026-8826129-s002.docx]

**Table S1. Baseline Characteristics of the Two Groups (x̄ ± s) (*n=50*).**

| **Variable** | **Observation Group (n=50)** | **Control Group (n=50)** | **P-value** |
| --- | --- | --- | --- |
| Male (n, %) | 32 (64.0%) | 33 (66.0%) | >0.05 |
| Female (n, %) | 18 (36.0%) | 17 (34.0%) | >0.05 |
| Age (years, mean ± SD) | 51.7 ± 6.4 | 52.2 ± 5.9 | >0.05 |
| Disease Duration (months, mean ± SD) | 1.4 ± 0.5 | 1.1 ± 0.3 | >0.05 |
